# Healthcare effects and evidence robustness of reimbursable digital health applications in Germany: a systematic review

**DOI:** 10.1038/s41746-025-01879-6

**Published:** 2025-08-01

**Authors:** Khira Sippli, Stefanie Deckert, Jochen Schmitt, Madlen Scheibe

**Affiliations:** Center for Evidence-Based Healthcare, Faculty of Medicine and University Hospital Carl Gustav Carus, TUD Dresden University of Technology, Dresden, Germany

**Keywords:** Outcomes research, Randomized controlled trials, Health care, Medical research

## Abstract

In Germany, statutory health insurance reimburses digital health applications (DiGAs) approved by the Federal Institute for Drugs and Medical Devices (BfArM). Permanent approval requires evidence of positive healthcare effects, either medical benefits or patient-relevant improvements in structures and processes (pSVV). This systematic review analyzed all 23 DiGA approval studies available as of March 15, 2024. The studies, conducted between 2012 and 2022, included 56 to 1245 participants and intervention durations from 1.5 to 12 months. Drop-out rates varied (mean 21.7% in intervention, 11.8% in control group). Most studies (13/23) focused on primary outcomes related to mental health conditions; one addressed pSVV. All reported significant medium to large effects, but risk of bias was high, particularly in outcome measurement (21/23) and due to missing data (15/23). These findings raise concerns about the robustness of the evidence supporting DiGA efficacy. The review recommends revising the DiGA approval process to enhance study quality. The systematic review was registered prospectively with PROSPERO https://www.crd.york.ac.uk/prospero/ (CRD42023460497).

## Introduction

With the adoption of the Digital Healthcare Act (DVG)^3^ in 2019, Germany became the first country in the world to systematically integrate digital health applications (DiGAs) into its statutory healthcare system, aiming to improve patient care and foster innovation. Since 2020, approved DiGAs can be prescribed to patients as a form of treatment, with the full cost reimbursed by their statutory health insurance. Unlike lifestyle apps, approved DiGAs are classified as medical devices, categorized as risk class I or IIa according to the Medical Device Regulation (MDR) or the Medical Device Directive (MDD); the latter was applicable under transitional regulations until the MDR came into full effect on May 26, 2021. With the introduction of the Digital Act (DigiG) in 2023, the scope was expanded to also allow certain medical devices that are categorized as risk class IIb to qualify as DiGA, provided that they are used in combination with remote medical supervision^1^.

DiGAs are specifically designed to support patient care in recognizing, monitoring, treating, or alleviating diseases, injuries, and disabilities^2^. The approval process for DiGAs is entirely new. Applications undergo a strict approval process by the Federal Institute for Drugs and Medical Devices (BfArM). After verifying the fulfillment of basic approval requirements, such as safety, functionality, data protection, information security, and interoperability of a DiGA, the BfArM assesses whether the manufacturer provides sufficient evidence for so-called positive healthcare effects of a DiGA and grants either provisional or permanent approval of a DiGA. Provisional approval is granted for DiGA of risk class I or IIa if preliminary evidence for positive healthcare effects is provided. The concept of positive healthcare effects was introduced by the Digital Healthcare Act (DVG)^3^, which categorized these as either medical benefits or patient-relevant improvements of structures and processes. Medical benefits encompass four categories of effects focused on improvements in a patient’s condition: improvement of health status, improvement of quality of life, reduction of disease duration, and prolongation of survival. Patient-relevant improvements of structures and processes encompass nine categories of effects focused on supporting the health behavior of patients or integrating processes between patients and healthcare providers. These categories are adherence, facilitating access to care, patient safety, health literacy, patient autonomy, coping with illness-related difficulties in everyday life, reduction of therapy-related expenses and burdens for patients and their relatives, alignment of treatment with guidelines and recognized standards, and coordination of treatment procedures^2^.

During a 12-month period of provisional approval, the manufacturer is obliged to provide further evidence of positive healthcare effects, otherwise, the approval is withdrawn. Under specific circumstances, the 12-month period may be extended by decision of the BfArM. If a manufacturer provides sufficient evidence of positive healthcare effects, permanent approval of the digital health application as DiGA is granted at the discretion of the BfArM. To be deemed sufficient evidence, approval studies must meet specific criteria outlined in the BfArM’s fast-track guidelines^2^. When doing this research and to date, the version of the fast-track guidelines dated December 28, 2023, is in effect. According to these fast-track guidelines, criteria to be met by approval studies include having at minimum a retrospective comparative study design, having a sufficient sample size (without specification), providing rates and reasons for drop-outs, being conducted in Germany, and having a comparison group oriented with the reality of healthcare^2^. After being granted permanent approval, manufacturers have 12 months to make their approval studies publicly available.

Thus, the DiGA approval procedure differs significantly from the established HTA process for assessing the benefits and harms of medicinal products in Germany, which is regulated by the German Medicines Market Reorganization Act (AMNOG)^4^. Under AMNOG, drugs that have demonstrated efficacy and safety, i.e. have a positive risk-benefit ratio, can be prescribed immediately upon market entry, but must subsequently undergo an additional benefit assessment. This assessment compares the new treatment with a standard therapy, a so-called appropriate comparative therapy, which is predefined by the Federal Joint Committee (G-BA), as well as outcomes for which the added benefit needs to be assessed. The process lasts six months and forms the basis for price negotiations, with the final price reflecting the assessed additional benefit. In contrast, the DiGA procedure does not involve predefinition of appropriate comparative therapies or outcomes by authorities. Moreover, the reimbursement price for DiGAs is not linked to the extent of additional benefit demonstrated. What both procedures share is the goal of making innovative and safe health interventions with a positive risk-benefit ratio rapidly accessible to patients.

Since the DiGA approval process is entirely new, there is ongoing discussion about the methodological quality of approval studies and the positive healthcare effects they demonstrate.

These discussions take place against the backdrop of a constantly evolving DiGA landscape, with new DiGA continuously receiving provisional or permanent approval, while others are removed. As of June 16, 2025, the DiGA directory^5^ lists 44 DiGAs with permanent and 14 DiGAs with provisional approval. Another 11 DiGAs had been delisted: six because no evidence of a positive healthcare effect could be provided, one because the clinical study for approval could not be completed, and four at the request of the manufacturer^5^. Since their introduction, the number of DiGA prescriptions has steadily increased, with over 1 million prescriptions issued by the end of 2024^6^. Approximately 81% of these were activated by patients, leading to cumulative expenditures of €234 million by the statutory health insurance funds between September 1, 2020, to December 31, 2024^6^. In 2024, the average price per DiGA prescription was €541, with prices per DiGA varying significantly, ranging from €119 to €2077 per use, which is typically limited to 90 days but in some cases extends up to 365 days^6^.

Despite these developments, knowledge about the overall risk of bias (RoB) of approval studies remains limited, as the BfArM does not provide public information on its evaluation of approval studies. Thus, it is unknown whether, how, and with which tools RoB assessments of approval studies are carried out by the BfArM as part of the DiGA approval process.

A comprehensive overview of the available published evidence for permanently approved DiGAs, therefore, appears both timely and necessary.

So far, two systematic reviews have conducted RoB assessment of a limited number of approval studies: Kolominsky-Rabas et al. assessed the approval studies for six permanently approved DiGAs from the categories “psychology” and “nervous system”^7^. Applying the revised Cochrane RoB tool, they found an overall high RoB^7^. Lantzsch et al.^8^ conducted a review and RoB assessment of eleven approval studies for eight of ten DiGAs that had been permanently approved as of February 2022. Using the same RoB tool as Kolominsky-Rabas et al., they also found an overall high RoB^8^. However, the RoB assessments for DiGA approval studies that were included in both these systematic reviews differed across several RoB domains.

Except for conducting a RoB assessment, both systematic reviews give no comprehensive overview of the characteristics of and evidence for positive healthcare effects measured in DiGA approval studies. It is, also in international comparison, a standard procedure to systematically review existing intervention studies on digital therapeutics and to compare their effects and their risk of bias using tools such as the RoB 2^9–14^.

Against this background, the two-fold objective of this systematic review was, for all DiGA approval studies that were published by March 15, 2024,

1. to provide a comprehensive overview of the characteristics of and evidence for positive healthcare effects,
2. to assess the RoB applying the revised Cochrane RoB tool (RoB 2)^15^

## Results

### Results of the search process

**Fig. 1:**
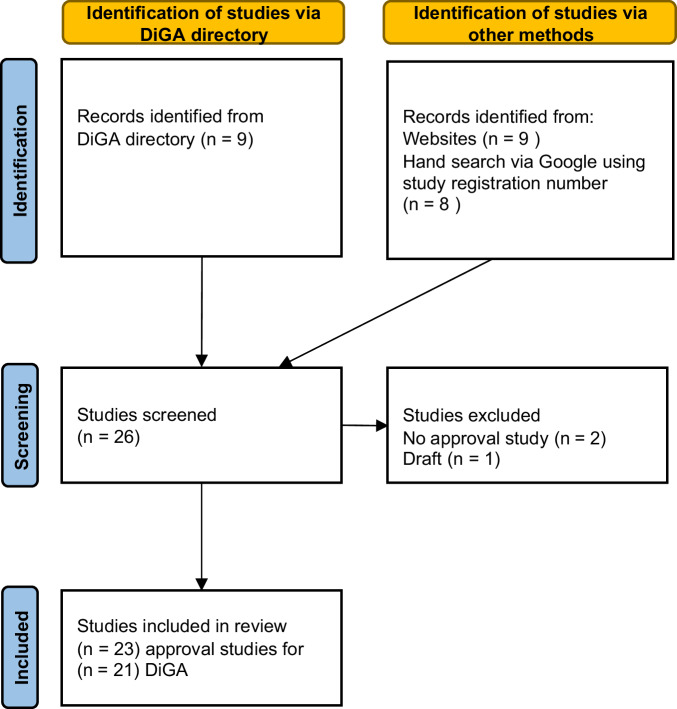
PRISMA flow chart. The figure outlines the screening and selection process of DiGA approval studies included in the systematic review, following the PRISMA 2020 guidelines.

Based on information in the DiGA directory for the 33 DiGAs that were permanently approved as of 15 March 2024, we were able to identify 23 approval studies for 21 DiGAs (Fig. 1). Two studies each were identified for the DiGAs deprexis and Kalmeda. For deprexis, the manufacturer listed two approval studies^16,17^ in the DiGA directory. For Kalmeda, a clinical study report^18^ was found, published on the manufacturer’s website in 2022, alongside a journal article^19^ named as an approval study on the manufacturer’s website in March 2024. Both were included in this review; the clinical study report^18^ had also been assessed in a previous review^8^.

Approval studies could not be identified for all 33 DiGAs, as manufacturers have 12 months to publish such studies after permanent approval is granted. As of March 15, 2024, this period had not yet expired for 12 permanently approved DiGAs, and the approval study had not been published yet. Of the identified 23 approval studies for 21 DiGA, nine were retrieved using the information on author, title, and the source provided by the DiGA directory (see Fig. 1). A further nine studies could be retrieved from the manufacturers’ websites, and the remaining five were identified via Google Scholar. Two additional studies were found via manual searches. These were excluded from the review, as it could not be verified that they were official approval studies. The participant numbers, details on outcomes, or study registration numbers of these studies did not match the information in the DiGA directory^20,21^. One identified study was an unpublished draft for a journal article; it was excluded due to the uncitable format and participant numbers that differed from the DiGA directory. In addition, 11 published study protocols^22–32^ corresponding to approval studies for 11 DiGAs were found via Google Scholar by applying the study registration numbers from DRKS or ClinicalTrials.gov, which are named in the DiGA directory for all approved DiGAs. The two approval studies included in this review for Kalmeda refer to the same study protocol^28^.

### Description of included studies

Table 1 depicts the characteristics of included approval studies. Data in this table are excerpts from the comprehensive data extraction sheet, available as Supplementary Data 1. Table 1 is available as an editable and filterable Excel file in Supplementary Data 2.

**Table 1:** Study and population characteristics of included studies (ordered by DiGA category, then alphabetically by DiGA name)

DiGA	DiGA category	Indication	Approval study (author, year)	Study design	Intervention description (intervention group)	Intervention duration (in months)	Control group intervention	Sample size	Sex (female) and age (mean)
Kalmeda	Ears	Other diseases of the ear, not elsewhere classified (H93.1)	Stover^18^	Parallel two-arm RCT	Self-guided, app-based intervention; based on cognitive behavioral therapy (CBT); exercises focusing on mindfulness, acceptance, self-efficacy, and relaxation; no individual customization; no reminder	9	Care as usual (CAU); access to DiGA after 3 months (waitlist)	*N* = 187 (IG: 94, CG: 93)	Sex female IG: 52.1% (*N* = 49), CG: 44.1% (*N* = 41); mean age: IG: 48.1 (SD 12.8), CG: 48.4 (SD 12.2)
Kalmeda	Ears	Other diseases of the ear, not elsewhere classified (H93.1)	Walter et al.^19^	Parallel two-arm RCT	Self-guided, app-based intervention; based on CBT, focusing on mindfulness, acceptance, self-efficacy and relaxation; no individual customization; no reminder	9	Care as usual (CAU); access to DiGA after 3 months (waitlist)	*N* = 187 (IG: 94; CG: 93)	Sex female: IG: 52.1% (N = 49), CG: 44,1% (*N* = 41); mean age: IG 48.1 (SD 12.8), CG 48.4 (SD 12.2)
Kranus Edera	Genitals, Kidneys, and Urinary Tract	Other diseases of the penis (N48.4)	Wiemer et al.^45^	Parallel two-arm RCT	Self-guided, app-based intervention; multidisclipinary program with daily and weekly exercises, including cardiovascular training, pelvic floor exercises, and psychoeducation modules (texts, videos); training tailored based on initial assessment; no reminder	3	Care as usual (CAU); access to DiGA after 3 months (waitlist)	*N* = 241 (IG: 122; CG: 119)	Sex female IG: 0% (*N* = 0), CG: 0% (*N* = 0); mean age IG: 53.76 (SD 13.62) CG: 49.74 (SD 12.73)
HelloBetter Diabetes	Hormones and Metabolism	Diabetes mellitus, type 1 (E10), Diabetes mellitus, type 2 (E11)	Balzus et al.^46^	Parallel two-arm RCT	Self-guided, web-based intervention; based on CBT techniques, including interactive modules (videos, audios, texts, exercises) and online diary; tailored based on individual progress; no reminder.	2	Access to DiGA after 12 months (waitlist)	*N* = 254 (IG: 128, CG: 126)	Sex female IG 63.3% (*N* = 81), CG 62.7% (*N* = 79); mean age: IG: 50.2 (SD 11.6), CG: 51.3 (SD 11.9)
Oviva Direkt für Adipositas	Hormones and Metabolism	Obesity (E66.00, E66.01)	Gemesi et al.^47^	Parallel two-arm RCT	Self-guided, app-based intervention; multi-modal weight loss program including modules for diet, physical activity, and behavioral change strategies; tailored based on individual weight loss goals and progress; automated progress feedback and reminder to enter data	3	Care as usual (CAU); access to DiGA after 3 months (waitlist)	*N* = 168 (IG: 84; CG: 84)	Sex female: IG 67.9% (*N* = 57), CG 60.7% (*N* = 51); mean age: IG 47.4 (SD 11.5), CG 46.3 (SD 10.6)
zanadio	Hormones and Metabolism	Obesity (E66.00, E66.01)	Roth et al.^48^	Parallel two-arm RCT	Self-guided, app-based intervention; multimodal weight loss approach combining nutrition, exercise, and behavioral therapy; tailored based on individual weight loss goals and progress; no reminder	12	Continuity of life routines, self-initiated weight loss activities allowed; access to DiGA after 3 months (waitlist)	*N* = 150 (IG: 76; CG: 73)	Sex female IG 90.7% (*N* = 69); CG: 91.8% (*N* = 67); mean age: IG 43.8 (SD 10.6), CG 42.9 (SD 11.1)
Kaia Rückenschmerzen	Muscles, Bones, and Joints	Back pain (M54)	Priebe et al.^34^	Cluster RCT	Self-guided, web-based intervention; multidisciplinary approach with educational content, physiotherapy, and mindfulness exercises, teleconsultation for patients at high risk of chronic back pain; no tailoring of content; no reminder	6	Standard treatment (according to national guidelines)	*N* = 1245 (IG: 933; CG: 312)	Sex female IG: 65% (*N* = 606); CG- 64% (*N* = 200); mean age: IG: 42 (SD 12.4); CG: 37 (SD 12.6)
Vivira	Muscles, Bones, and Joints	Osteochondrosis of the spine (M42.0, M42.1, M42.9), other diseases of the spine and back, not elsewhere classified (M53.2, M53.8, M53.9), back pain (M54.4, M54.5, M54.6, M54.8, M54.9), biomechanical dysfunctions, not elsewhere classified, Segmental and somatic dysfunctions (M99.02, M99.03, M99.04), Other biomechanical dysfunctions (M99.82, M99.83, M99.84)	Weise et al.^49^	Parallel two-arm RCT	Self-guided, app-based intervention; based on movement therapy and functional regional interdependence; multimodal approach (video, audio, text) with daily movement exercises; adaptation of exercise selection based on user feedback; no reminder	3	Standard physiotherapy treatment according to guidelines; access to DiGA after 3 months (waitlist)	*N* = 215 (IG: 108; CG: 107)	Sex female IG 47.2% (*N* = 51), CG: 59.1% (N = 62); mean age: IG: 57.4 (SD 13.8), CG: 57.3 (SD 13.5)
elevida	Nervous System	Multiple sclerosis [Encephalomyelitis disseminata] (G35)	Pöttgen et al.^50^	Parallel two-arm RCT	Self-guided, web-based intervention; based on CBT; simulated dialogs and exercises to manage fatigue; content dynamically adjusted based on user responses; no reminder	3	Care as usual (CAU); access to DiGA after 6 months (waitlist)	*N* = 275 (IG: 139, CG: 136)	Sex female IG: 82% (*N* = 114); CG: 79% (*N* = 108); mean age: IG: 40.8 (SD 11.1); CG: 41.9 (SD 9.4)
HelloBetter Stress and Burnout	other	Problems related to difficulties in coping with life (Z73)	Heber et al.^51^	Parallel two-arm RCT	Self-guided, web-based intervention; based on Lazarus´ transactional model of stress, applying problem solving and emotional regulation strategies; exercises with interactive elements (audio, video, downloads); tailored based on individual progress; non-therapeutic feedback after session completion and optional mobile reminders	1.75	Care as usual (CAU); access to DiGA after 1.75 months (waitlist)	*N* = 264 (IG: 132, CG: 132)	Sex female IG 73,5% (*N* = 97), CG 72.7% (*N* = 96); mean age: IG: 42.4 (SD 10.7), CG: 44.2 (SD 9.6)
deprexis	Psychology	Depressive episode (F32.0, F32.1, F32.2), recurrent depressive disorder (F33.0, F33.1, F33.2)	Klein et al.^16^	Parallel two-arm RCT	Self-guided, web-based intervention; based on CBT technique including cognitive restructuring, mindfulness, acceptance, and behavioral activation; engaging users in interactive exercises, daily symptom tracking, and feedback; tailored based on feedback; daily messages via SMS or e-mail, and reminder after no use for 2 weeks	3	Care as usual (CAU); access to DiGA after 6 months (waitlist)	*N* = 1.013 (IG: 509, CG: 504)	Sex female: IG 68,8% (*N* = 350), CG 68.5% (*N* = 345); mean age: IG 42.8 (SD: 11); CG 42.9 (SD 11)
deprexis	Psychology	Depressive episode (F32.0, F32.1, F32.2), recurrent depressive disorder (F33.0, F33.1, F33.2)	Meyer et al.^17^	Parallel two-arm RCT	Self-guided, web-based intervention consistent with CBT; offering simulated dialog including cognitive restructuring, behavioral activation, mindfulness, problem solving, positive psychology; engaging users in exercises with worksheets, summary sheets, audio-recordings, photos, illustrations, and symptom tracking; content tailored following feedback; optional daily reminder per sms or e-mail	3	Care as usual (CAU); access to DiGA after 6 months (waitlist)	*N* = 163 (IG: 78; CG: 85)	Sex female: IG: 74.4% (*N* = 58), CG: 75.3% (*N* = 64); mean age: IG: 44 (SD 11.02); CG: 40 (SD 11.48)
HelloBetter Panik	Psychology	Phobic disorders (F40.01), other anxiety disorders (F41.0)	Ebenfeld et al.^37^	Parallel two-arm RCT	Guided, web- and app-based intervention; offering CBT training, including psychoeducation, cognitive restructuring, relaxation, and exposure exercises; no tailored content; written feedback by coach after every session to increase adherence and motivation; reminder sent by coaches if no login for 1 week	2	Access to DiGA after 6 months (waitlist)	*N* = 92 (IG: 45, CG: 47)	Sex female IG 60% (*N* = 27); CG: 51% (*N* = 24); mean age IG 39.33 (SD 10.83), CG 37.43 (SD 10.03)
HelloBetter Vaginismus Plus	Psychology	Sexual dysfunction not caused by an organic disorder or disease (F52.5, F52.6)	Zarski et al.^35^	Parallel two-arm RCT	Self-guided, web-based intervention; based on CBT for GPPPD; including cognitive restructuring, psychoeducation, mindfulness, relaxation, strengthening dyadic coping; tailored content based on user feedback; motivational text messages and practice reminders by eCoach if module was not completed in 7 days, semi-structured personalized written feedback on session by eCoach	8 sessions	Care as usual (CAU); access to DiGA after 6 months (waitlist)	*N* = 200 (IG: 100, CG: 100)	Sex female: IG 100% (*N* = 100), CG 1005 (*N* = 100); mean age: IG 29.46 (SD 9.82); CG: 28.04 (SD 7.84)
Invirto-die Therapie gegen Angst: agoraphobia	Psychology	Phobic disorders (F40.00, F40.01)	Zurowski et al.^38^	Parallel two-arm RCT	Guided, app-based, virtual reality intervention; based on CBT for anxiety disorder, including exposure exercises; 2 video-telephone contacts with therapists; content disorder-specific but not tailored; daily reminders via SMS and e-mail	Self-paced, ~2 months; optional 4 additional months	Active control with supportive contacts every 4 weeks; care as usual (CAU); access to DiGA after 6 months (waitlist)	Agoraphobia: *N* = 103 (IG: 72, CG: 31)	Sex female: IG:62.5% (*N* = 45), CG: 80.7% (*N* = 2); mean age: IG: 35.8 (SD 9.4), CG: 36.3 (SD 10.5)
Invirto-die Therapie gegen Angst: panic disorder	Psychology	Other anxiety disorders (F41.0)	Zurowski et al.^38^	Parallel two-arm RCT	Guided, app-based, virtual reality intervention; based on CBT for anxiety disorder, including exposure exercises; 2 video-telephone contacts with therapists; content disorder-specific but not tailored; daily reminders via SMS and e-mail	Self-paced, ~2 months; optional 4 additional months	Active control with supportive contacts every 4 weeks; care as usual (CAU); access to DiGA after 6 months (waitlist)	Panic disorder *N* = 84 (IG: 58, CG: 26)	Sex female: IG:62,1% (*N* = 36), CG: 69.2% (*N* = 18); mean age: IG: 37 (SD 12), CG: 36.5 (SD 12.3)
Invirto-die Therapie gegen Angst: social phobia	Psychology	Phobic disorders (F40.1)	Zurowski et al.^38^	Parallel two-arm RCT	Guided, app-based, virtual reality intervention; based on CBT for anxiety disorder, including exposure exercises; 2 video-telephone contacts with therapists; content disorder-specific but not tailored; daily reminders via SMS and e-mail	Self-paced, ~2 months; optional 4 additional months	Active control with supportive contacts every 4 weeks; care as usual (CAU); access to DiGA after 6 months (waitlist)	Social phobia *N* = 110 (IG: 67, CG: 43)	Sex female: IG: 52.2% (*N* = 35), CG: 58.1% (*N* = 25); mean age: IG:32,36 (SD 9.91), CG: 32.9 (SD 9,6)
Mindable: Panik und Agoraphobie	Psychology	Phobic disorders (F40.0), Other anxiety disorders (F41.0)	Helbig-Lang et al.^39^	Parallel two-arm RCT	Self-guided, app-based intervention; based on exposure therapy; including psychoeducation, symptom monitoring (diary), and exposure exercises; exercises adjusted according to patients´ anxiety levels and feedback in app, users can create and modify their own exposure exercises; weekly check-ups based on DSM-5 to increase engagement	2	Standard self-help recommendations during the waiting period, permission to seek external help such as psychotherapy; not reported whether access to DiGA was granted after waiting period	*N* = 107 (IG: 57; CG: 50)	Sex female IG 71,9% (*N* = 41), CG: 82% (*N* = 41); mean age: IG: 36.35 (SD 14.4), CG:35.82 (SD 14.17)
NichtraucherHelden-App	Psychology	Mental and behavioral disorders caused by tobacco (F17.2)	Rupp et al.^40^	Parallel two-arm RCT	Self-guided, app-based intervention; based on smoking cessation strategies, offering behavioral support and structured modules including progress tracking; personalized support and guidance based on user´s progress and needs; no reminder	Duration of intervention not clear: no start date set; 9 core units plus optional 76-day follow-up care	Brief advice yet no treatment, patients asked to use no other app or support; access to DiGA after end of study (waitlist)	*N* = 661 (IG: 336; CG: 325)	Sex female: IG: 61.2% (*N* = 205); CG: 61.8% (*N* = 197), mean age: IG 46 (SD 12), CG 46 (SD 12)
Novego Depressionen bewältigen	Psychology	Depressive episode (F32.0, F32.1, F32.2), Recurrent depressive disorder (F33.0, F33.1, F33.2), persistent affective disorder (F34.1)	Baumeister and Moritz^42^	Parallel two-arm RCT	Self-guided, web-based intervention; based on CBT, system therapy, mindfulness therapy; interactive exercises (text, audio, video, illustrations) on psychoeducation, and exercises; modules compiled from pool based on initial questionnaire, but standardized CBT content; e-mail or sms reminder, written support for questions, and crisis hotline	3	Care as usual (CAU); access to DiGA after 6 months (waitlist)	*N* = 303 (IG: 153; CG: 150)	Sex female: IG: 69.9% (*N* = 107); CG: 74% (*N* = 74); mean age: IG: 40.82 (SD 11.91), CG: 40.54 (SD 13.11)
Selfapys Online Kurs bei Depression	Psychology	Depressive episode (F32.0, F32.1), recurrent depressive disorder (F33.0, F33.1)	Krämer et al.^33^	three-arm RCT	Unguided or self-guided, web-based intervention; based on CBT, systemic therapy, mindfulness training; engaging users in active exercises, mood trajectory; personalized feedback, individual goals can be set and reviewed; optional reminder e-mails and chat support to clarify questions; in guided group, personal guidance by psychotherapists (weekly telephone calls) to discuss exercises	3	Weekly standardized mindfulness exercises via email, with content comparable to that of a self-help mindfulness guide; permission to seek pharmacological/psychological treatments in waiting time (CAU); access to intervention after 6 months	*N* = 401 (IG1 *N* = 151, IG2 *N* = 150, CG *N* = 100)	Sex female: IG1: 83.4% (*N* = 126); IG2: 84% (*N* = 126); CG: 81% (*N* = 81); mean age: IG1: 38 (SD 10.7) IG2: 37 (SD 10.8) CG: 36 (SD 11.9)
Selfapys Online-Kurs bei Generalisierter Angststörung	Psychology	Other anxiety disorders (F41.1)	Rubel et al.^41^	Parallel two-arm RCT	Self-guided, web-based intervention; based on CBT and mindfulness-based therapy; including interactive mindfulness, exposure, and problem-solving exercises; in-depth modules individually selectable based on users' needs; no reminder	3	Care as usual (CAU); access to DiGA after 3 months (waitlist)	*N* = 156 (IG: 78; CG: 78)	Sex female: IG: 88.5% (*N* = 69), CG: 75.6% (*N* = 59); mean age IG: 33.2 (SD 10.5), CG: 37.2 (SD 12.4)
somnio	Psychology	Non-organic sleep disorders (F51.0), sleep disorders (G47.0)	Lorenz et al.^43^	Parallel two-arm RCT	Self-guided, web-based intervention; based on CBT for insomnia; including modules for sleep continuity, sleep quality and daytime performance, sleep diary; self-paced with feedback on progress on individual goals; tailored dialog with animated sleep coach based on individual response behavior; e-mail reminder if sleep diary is not filled in, continuing with program conditional on sleep diary completion	1.5	Care as usual (CAU); access to DiGA after 1.5 months (waitlist)	*N* = 56 (IG: 29; CG: 27)	Sex female IG: 72% (*N* = 21), CG: 67% (*N* = 18); mean age: IG: 41.72 (SD 17.31), CG: 44.04 (SD 20.05)
velibra	Psychology	Phobic disorders (F40.01, F40.1), other anxiety disorders (F41.0, F41.1)	Berger et al.^44^	Parallel two-arm RCT	Self-guided, web- and app-based intervention; based on CBT emphasizing transdiagnostic principles; including psychoeducation, cognitive-behavioral and anxiety management exercises, symptom tracking; content tailored based on user responses to symptom tracking questions; daily diary tasks and reminders via SMS or email to support engagement.	2.25	Care as usual (CAU); access to DiGA after 9 weeks (waitlist)	*N* = 139 (IG: 70; CG : 69)	Sex female IG 69% (*N* = 48); CG 72% (*N* = 40); mean age: IG: 42.1 (SD 12.2), CG: 41.8 (SD 12.2)
vorvida	Psychology	Mental and behavioral disorders due to alcohol (F10.1, F10.2)	Zill et al.^36^	Parallel two-arm RCT	Self-guided, web-based intervention; based on CBT; including psychoeducation, self-monitoring and behavioral strategies, mood checker; program adapts based on user input and behavior (simulated dialog); daily reminder via SMS	6	Care as usual (CAU); access to DiGA after 6 months (waitlist)	*N* = 608 (IG: 306; CG: 302)	Sex female IG: 56% (*N* = 170); CG: 49% (*N* = 149); mean age: IG: 40.4 (SD 11.2); CG: 40.7 (SD: 12.1)

This table provides an overview of key characteristics of the 23 DiGA approval studies included in this review. The studies are ordered by DiGA category according to the BfArM DiGA directory, followed alphabetically by DiGA name.

Balzus et al.^46^: The DiGA “HelloBetter Diabetes” was renamed in 2024; the former name was “HelloBetter Diabetes und Depression”.

Ebenfeld et al.^37^: It was not reported whether care-as-usual was permitted/forbidden for the control group.

The approval studies for all identified DiGAs were conducted between August 2012 and February 2022. For 21 of the approval studies, the study design was a parallel, two-arm randomized controlled trial. One study was a three-arm randomized controlled trial^33^, and one was a cluster-randomized controlled trial^34^. The majority of approval studies—13 of 23—included in this systematic review are from the DiGA directory category “psychology.”^16,17,33,35–44^ Other DiGA directory categories for which approval studies were identified are “ears,”^18,19^ “genitals, kidneys and urinary tract,”^45^ “hormones and metabolism,”^46–48^ “muscles, bones and joints,”^34,49^ “nervous system,”^50^, and “other”^51^. The typical intervention was self-guided, web-based, and included elements from cognitive behavioral therapy (CBT), with content delivered through interactive exercises and multimedia.

In one study^38^, the intervention group was composed of sub-groups of participants with three different indications. The intervention was tailored in an indication-specific manner, and outcomes were measured separately. Thus, the approval study was counted as three interventions, and information is listed for each group separately in the tables.

Reminders for maintaining motivation and adherence were applied in 11 interventions, and were optional in three others. The intervention duration ranged from 1.5 to 12 months, with a mean duration of 3.8 months. The control group received care-as-usual in 21 studies, with access provided to the DiGA after a mean waiting period of 5.0 months. In two studies, participants of the control group were never given access to the DiGA^34,39^.

Sample sizes varied widely, with the number of participants ranging from 56 to 1245, with a mean of *N* = 321 (intervention and control groups combined). The majority of study participants were female, ranging from 47.2% to 90.7% (mean 68.8%) in the intervention groups (excluding two studies for DiGA that were designed exclusively for women^35^ or men^45^, respectively. The mean age of the intervention groups ranged from 29.4 to 57.4 years (mean 42.1 years). In control groups, the share of female participants ranged from 44.1% to 91.8% (mean 67.3%; excluding the two approval studies for DiGA that were designed exclusively for women^35^ or men^45^, respectively. The mean age in the control groups ranged from 28.0 to 57.3 years (mean 41.7 years).

### Overview of outcomes and effects

Table 2 shows the primary outcomes, measurement time points, and measurement instruments used in the studies, as well as the effects and effect sizes found (available as an editable and filterable Excel file in Supplementary Data 3). Information on secondary outcomes is contained in the data extraction sheet (see Supplementary Data 1).

**Table 2:** Primary outcomes and effects of interventions (ordered by DiGA category, then alphabetically by DiGA name)

DiGA	DiGA category	Approval study (author, year)	Primary Outcome(s)	Rationale for primary outcome(s)	Outcome domain (domains of mN/psVV)	Outcome Measurement instruments (OMIs)	Outcome measurement patient self-reported	Intervention duration (in months)	Timing of post-intervention measurement (intervention duration in months)	Drop- out until post-intervention	Number of follow-ups (distance from baseline in months)	Significant between group effect at post-assessment	Between-group effect size at post-assessment	effect size according to study authors
Kalmeda	Ears	Stover^18^	Tinnitus exposure	Implicit: improvement of care; implicit: high burden or impact on quality of life	Medical benefit: improvement of health	Tinnitus overall score as measured by Tinnitus Questionnaire (TQ) (Hiller/Göbel 2004)	Yes	9	During intervention (3 months)	IG: 16/94 = 17.1%; CG: 8/93 = 8.6% (without App usage)	1 (9 months)	Yes	d calculated but not reported	N/A
Kalmeda	Ears	Walter et al.^19^	Tinnitus distress	Implicit: high prevalence of condition	Medical benefit: improvement of health	Tinnitus Questionnaire (TQ) (Göbel/Hiller 1998)	Yes	9	During intervention (3 months)	IG: 16/94 = 17.1%; CG: 8/93 = 8,6%	1 (9 months)	Yes	Reduction *d* = 1.1 (*p* < 0.001)	Large
Kranus Edera	Genitals, Kidneys, and Urinary Tract	Wiemer et al.^45^	Improvement of erectile dysfunction	Implicit: clinical relevance/core symptom; implicit: high prevalence of condition; implicit: high burden or impact on quality of life	Medical benefit: improvement of health	5 item version of International Index of Erectile Function (IIEF-5) (Rosen, 1997, 1999); German version (Wiltink 2003)	Yes	3	At end of intervention (3 months)	IG: 5/122 = 4%; CG: 1/119 = 1%	No follow-up	Yes	Improvement *d* = 1.36 (*p* < 0.0001)	Large
Kranus Edera	Genitals, Kidneys, and Urinary Tract	Wiemer et al.^45^	Improvement in disease-related quality of life	Implicit: clinical relevance/core symptom; implicit: high prevalence of condition; implicit: high burden or impact on quality of life	Medical benefit: improvement of quality of life	QoL-Med (Wagner et al. 1996), German version (Ruof et al. 2001)	Yes	3	At end of intervention (3 months)	IG: 5/122 = 4%; CG: 1/119 = 1%	No follow-up	Yes	Improvement *d* = 1.47 (*p* < 0.0001)	Large
Kranus Edera	Genitals, Kidneys, and Urinary Tract	Wiemer et al.^45^	Improvement of patient sovereignty/ activity	Implicit: clinical relevance/core symptom; implicit: high prevalence of condition; implicit: high burden or impact on quality of life	Patient-relevant structural and procedural improvements: patient sovereignty	PAM-13 (Hibbard et al. 2004), German version (Brenk et al. 2013)	Yes	3	At end of intervention (3 months)	IG: 5/122 = 4%; CG: 1/119 = 1%	No follow-up	Yes	Improvement *d* = 0.96 (*p* < 0.0001)	Medium to large
HelloBetter Diabetes	Hormones and Metabolism	Balzus et al.^46^	Depressive symptoms	Implicit: clinical relevance/core symptom; implicit: high burden or impact on quality of life	Medical benefit: improvement of health	Allgemeine Depressionsskala (ADS) (Hautzinger et al. 2012 a,b); Schmitt et al 2013); (engl.: CES-D, Radloff 1977; Vilagut 2016)	Yes	2	At end of intervention (2 months)	IG: 31/130 = 23.8%; CG: 16/130 = 12.3%	2 (6 months and 12 months)	Yes	Reduction *d* = 0.94 (*p* < 0.001)	Large
Oviva Direkt für Adipositas	Hormones and Metabolism	Gemesi et al.^47^	Weight change	Implicit: high prevalence of condition; implicit: high burden or impact on quality of life	Medical benefit: improvement of health	BMI (body height, body weight), body composition (bioimpedance analysis scale) (Gemesi et al.^47^)	No: objective measurement by study team	3	At end of intervention (3 months)	IG: 21/84 = 25%; CG: 8/84 = 9.5%	1 (6 months)	Yes	Improvement difference of 2.9 kg in weight loss between IG and CG (beta = −2.9, 95% CI: −3.8;−1.9) (*p* < 0.001); standardized regression coefficient: 0.45–0.46 (*p* < 0.001)	Not reported
zanadio	Hormones and Metabolism	Roth et al.^48^	Weight change	Implicit: high prevalence of condition	Medical benefit: improvement of health	Percentage weight change from baseline (T0) to 12 months (T4) (Roth et al.^48^)	Yes: self-measured by participants	12	At end of intervention (12 months)	IG: 9/77 = 11.7%; CG: 6/73 = 8.2%	No follow-up	Yes	Weight loss; *d* = −1.11 (*p* = 0.003)	Not reported
Kaia Rückenschmerzen	Muscles, Bones, and Joints	Priebe et al.^34^	Pain intensity	Implicit: high prevalence of condition; implicit: supported by Meta-Analysis or Research Evidence	Medical benefit: improvement of health	11 point numerical pain score (NRS) (Dworkin et al. 2005; Chiarotto et al. 2015)	Yes	6	At end of intervention (6 months)	IG: 253/933 = 27.2%; CG; 51/312 = 6.4% CG	2 (timing not reported)	Yes	Reduction 33.3% (IG) vs. –14.3% (CG) (*p* < 0.001)	Not reported
Vivira	Muscles, Bones, and Joints	Weise et al.^49^	Back pain	Implicit: high burden or impact on quality of life	Medical benefit: improvement of health	Verbal numerical rating scale (vNRS) for nonmalignant pain (Aicher et al. 2012)	Yes	3	At end of intervention (3 months)	IG: 10/108 = 9.2%; CG: 6/107 = 5.6%	No follow-up	Yes	Reduction Mean difference in pain score = −2.44 (95%CI: −2.92; −1,95) (*p* < 0.001)	Not reported
Elevida	Nervous System	Pöttgen et al.^50^	Severity of physical and mental fatigue	Implicit: clinical relevance/core symptom	Medical benefit: improvement of health	Chalder Fatigue Scale (Cella/Chalder 2010)	Yes	3	At end of intervention (3 months)	IG: 36/139 = 25.8%; CG: 15/136 = 11%	1 (6 months)	Yes	Reduction *d* = 0.53 (*p* < 0.0007)	Medium
HelloBetter Stress and Burnout	other	Heber et al.^51^	Stress exposure (perceived stress)	Implicit: high prevalence of condition	Medical benefit: improvement of health	Perceived Stress Scale PSS-10 (Cohen et al. 1983, 2006, 2009)	Yes	1.75	At end of intervention (7 weeks)	IG: 16/132 = 12.1%; CG: 5/132 = 3.8%	2 (6 months and 12 months)	Yes	Reduction d = 0.83 (*p* < 0.001)	Large
Deprexis	Psychology	Klein et al.^16^	Depressive symptom severity	Implicit: improvement of care	Medical benefit: improvement of health	PHQ-9 (Kroenke et al., 2001)	Yes	3	At end of intervention (3 months)	IG: 114/509 = 22%, CG: 105/504 = 21%	1 (6 months)	Yes	Reduction *d* = 0.39 (*p* < 0.001)	Small to medium
Deprexis	Psychology	Meyer et al.^17^	Depressive symptom severity	Implicit: improvement of care	Medical benefit: improvement of health	PHQ-9 (Gräfe et al., 2004; Kroenke et al., 2001; Löwe et al., 2004; Martin et al., 2006)	Yes	3	At end of intervention (3 months)	IG: 17/78 = 21.8%; CG: 12/85 = 14.1%	1 (6 months)	Yes	Reduction *d* = 0.57 (*p* < 0.01)	Medium
HelloBetter Panik	Psychology	Ebenfeld et al.^37^	Severity of panic and agoraphobia symptoms	Implicit: high prevalence of condition	Medical benefit: improvement of health	Panic and Agoraphobia Scale (PAS) (Bandelow 1995, 1997, 2000), German version: Panik- und Agoraphobieskala	Yes	2	At end of intervention (2 months)	IG 4/45 = 8.8%, CG 4/47 = 8.5%	2 (3 months and 6 months)	Yes	Reduction *d* = 0.66 (*p* = 0.002)	Medium to large
HelloBetter Vaginismus Plus	Psychology	Zarski et al.^35^	Intercourse penetration behavior	Implicit: high burden or impact on quality of life	Medical benefit: improvement of health	PEQ (van Lankveld. 2006)	Yes	8 sessions	At end of intervention (completion of session 8 or 3 months)	IG: 22/100 = 22%; CG: 8/100 = 8%	1 (6 months)	Yes	Improvement OR = 3.01 (95% CI: 1.46–6.18); (*p* < 0.01)	Not reported
Invirto-die Therapie gegen Angst: agoraphobia	Psychology	Zurowski et al.^38^	Reduction of symptoms of anxiety	Implicit: high prevalence of condition; implicit: high burden or impact on quality of life	Medical benefit: improvement of health	Becks Anxiety Inventory (BAI) (Margraf and Ehlers, 2007)	Yes	Self-paced, approx. 2 months; optional 4 additional months	At end of intervention (2 months)	22/103 = 21% (IG: 29.5%, CG: 3.2%)	2 (6 months and 12 months)	Yes	Reduction *d* = 0.52 (*p* not reported)	Medium
Invirto-die Therapie gegen Angst: panic disorder	Psychology	Zurowski et al.^38^	Reduction of symptoms of anxiety	Implicit: high prevalence of condition; implicit: high burden or impact on quality of life	Medical benefit: improvement of health	Becks Angst Inventar (BAI) (Margraf and Ehlers, 2007)	Yes	Self-paced, approx. 2 months; optional 4 additional months	At end of intervention (2 months)	27/84 = 32.1% (IG: 43.1%, CG: 7.6%)	2 (6 months and 12 months)	Yes	Reduction *d* = 0.50 (*p* not reported)	Medium
Invirto-die Therapie gegen Angst: social phobia	Psychology	Zurowski et al.^38^	Reduction of symptoms of anxiety	Implicit: high prevalence of condition; implicit: high burden or impact on quality of life	Medical benefit: improvement of health	Becks Angst Inventar (BAI) (Margraf and Ehlers, 2007)	Yes	Self-paced, approx. 2 months; optional 4 additional months	At end of intervention (2 months)	36/110 = 32.7% (IG: 44.7%, CG: 13.9%)	2 (6 months and 12 months)	Yes	Reduction *d* not reported, (p not reported)	Large
Mindable: Panik und Agoraphobie	Psychology	Helbig-Lang et al.^39^	Panic and agoraphobia symptoms	Implicit: high prevalence of condition; implicit: high burden or impact on quality of life	Medical benefit: improvement of health	Panic and agoraphobia scale (PAS) (Bandelow, 2016)	Yes	2	At end of intervention (2 months)	IG: 9/57 = 15.7%; CG: 1/50 = 2%	No follow-up	Yes	Reduction *d* = 0,7 (*p* < 0.001)	N/A
Nichtraucherhelden-App	Psychology	Rupp et al.^40^	Self-reported abstinence from tobacco products	Implicit: clinical relevance/core symptom; implicit: high prevalence of condition	Medical benefit: improvement of health	Self-reported abstinence from tobacco products (7-day point prevalence)	Yes	Duration of intervention not clear: no start date set; 9 core units plus optional 76 day follow-up care	At end of intervention (6 months)	IG 180/336 = 53.5%; CG 105/325 = 32.3% (until t3 = 3 M)	No follow-up	Yes	Improvement 20.2% in IG vs. 10.5% in CG at t4 OR = 2.2 (*p* < 0.001)	Not reported
Novego Depressionen bewältigen	Psychology	Baumeister and Moritz^42^	Change of severity of depressive symptoms	Implicit: clinical relevance/core symptom; implicit: high burden or impact on quality of life	Medical benefit: improvement of health	BDI-II (Beck, Steer and Brown, 1996)	Yes	3	At end of intervention (3 months)	IG: 47/153 = 30.7%; CG: 37/150 = 24.7%	No follow-up	Yes	Reduction Hedge´s *g* = 0.304 (*p* = 0.017)	Small to medium
Selfapys Online-Kurs bei Depression	Psychology	Krämer et al.^33^	Depressive symptoms	Implicit: clinical relevance/core symptom of Condition; implicit: supported by meta-analysis or research evidence	Medical benefit: improvement of health	Beck Depression Inventory score (BDI-II) (Beck 2013)	Yes	3	At end of intervention (3 months)	guided: 19/151 = 12.5%; unguided: 34/150 = 22.6%; CG: 47/100 = 47%	1 (6 months)	Yes	Reduction *d* = 1.63 : guided vs. control group (*p* < 0.001) *d* = 1.47 unguid-ed vs. control group (*p* < 0.001)	Large
Selfapys Online-Kurs bei Generalisierter Angststörung	Psychology	Rubel et al.^41^	Generalized anxiety disorder symptoms	Implicit: high prevalence of condition	Medical benefit: improvement of health	Generalized Anxiety Disorder 7 (GAD-7) (Spitzer et al. 2006)	Yes	3	At end of intervention (3 months)	IG: 18/8 = 23.1%; CG: 6/78 = 7.7%	No follow-up	Yes	Improvement *d* = 0.88 (*p* < 0.001)	Large
Selfapys Online-Kurs bei Generalisierter Angststörung	Psychology	Rubel et al.^41^	Well-being	Implicit: high prevalence of condition	Medical benefit: improvement of quality of life	WHO Wellbeing Index-5 (WHO-5) (Topp et al. 2015)	Yes	3	At end of intervention (3 months)	IG: 18/8 = 23.1%; CG: 6/78 = 7.7%	No follow-up	Yes	Increase *d* = 0.62 (*p* < 0.001)	Medium to large
Somnio	Psychology	Lorenz et al.^43^	Insomnia severity	Implicit: high burden or impact on quality of life	Medical benefit: improvement of health	Insomnia severity index (ISI) (Bastien et al., 2001)	yes	1.5	At end of intervention (1.5 months)	IG: 4/29 = 7%; CG: 27/27 = 0%	1 (12 months)	Yes	Improvement *d* = 1.79 (*p* < 0.001)	Large
Velibra	Psychology	Berger et al.^44^	Reduction of anxiety and related symptoms among patients with social anxiety disorder, panic disorder with/without agoraphobia, and/or generalized anxiety disorder	Implicit: high prevalence of condition; implicit: supported by meta-analysis or research evidence	Medical benefit: improvement of health	Disorder unspecific measures of anxiety, depressive symptoms, tension/stress: BAI (Beck et al. 1988) BDI-II (Beck et al. 1996) DASS-21 (Lovibond & Lovibond, 1995) BSI (Derogatis, 1993) using the Global Severity Index (GSI)	yes (all but SCID-I)	2.25	At end of intervention (2.25 months)	IG: 13/70 = 19%; CG: 6/69 = 9%	1 (6 months)	Yes	Reduction BAI: *d* = 0.41 (*p* < 0.001) BDI-II: *d* = 0.61 (*p* < 0.001) BSI-GSI: *d* = 0.42 (*p* < 0.001) DASS-21: *d* = 0.47 (*p* < 0.01) = small to medium effects	Small to medium (BAI, BSI-GSI, DASS); medium to large (BDI-II)
Velibra	Psychology	Berger et al.^44^	Quality of life	Implicit: high prevalence of condition; implicit: high burden or impact on quality of life	Medical benefit: improvement of quality of life	SF-12 mental (Ware et al 1996)	yes	2.25	At end of intervention (2.25 months)	IG: 13/70 = 19%; CG: 6/69 = 9%	1 (6 months)	Yes: SF-12 MH	Improvement SF12-MH: *d* = 0.49 (*p* < 0.001)	Small to medium
Vorvida	Psychology	Zill et al.^36^	Reduction of alcohol consumption	Implicit: high prevalence of condition; implicit: high burden or impact on quality of life	Medical benefit: improvement of health	Self-reported alcohol consumption in last 30 or 7 days according to Quantity-Frequency-Index (QFI) (Bloomfield et al. 2013; Kraus et al. 2013) and „timeline followback“-method (TFB) (Sobell/Sobell 1992) (Zill et al.^36^)	Yes	6	At end of intervention (6 months)	IG: 114/306 = 37.2%, CG: 69/303 = 22.8%	No follow-up	Yes	Reduction QFI: *d* = 0.34 (*p* < 0.001) TFB: *d* = 0.54 (*p* < 0.001)	Small to medium (QFI); medium (TFB)

This table summarizes key characteristics and findings of the 23 DiGA approval studies included in this review. The studies are ordered by DiGA category according to the BfArM DiGA directory, followed alphabetically by DiGA name.

The 23 approval studies included between one and three primary outcomes, totaling 29 across all studies (mean: 1.2; median: 1). In addition, the studies examined between 0 and 13 secondary outcomes (total: 120; mean: 4.8; median: 4) and 0–6 other outcomes (total: 25; mean: 1 median: 0). The categorization of outcomes as “primary”, “secondary” or “other” (including “explorative”/“additional”/“other”) were extracted from the studies themselves. In counting outcomes, the study by Zurowski et al.^38^ was recorded three times, as the intervention was tailored to three distinct indications of participants in the intervention group, and these three interventions were assessed separately.

The 29 primary outcomes, which are decisive for the permanent approval of a DiGA, were assigned to different outcome domains within either the “medical benefit” or “patient-relevant improvements of structures and processes” categories. As some of the sub-categories are new in terms of proof of benefit and are not comprehensively defined by the BfArM fast-track guidelines, the assignment was performed according to the best knowledge of the authors of this systematic review. Of the 29 primary outcomes, 28 were assigned to the “medical benefit” outcome domain, specifically within the subcategories of “improvement of health” (25) or “improvement of quality of life” (3). One primary outcome was assigned to the “patient-relevant improvements of structures and processes” outcome domain, specifically within the subcategory “improvement of patient autonomy.”^45^

The choice of the primary outcomes was not explicitly justified in the approval studies. Thus, the implicit rationales were identified for all primary outcomes as part of the review. For 12 primary outcomes, only one rationale was identified; for 14 primary outcomes, two rationales were identified; and for three primary outcomes, three rationales were assigned. Thus, a total of 49 implicit rationales were assigned to the 29 primary outcomes. Implicit rationales identified were classified according to the categories in the methods section: high prevalence of condition (*n* = 19), high burden or impact on quality of life (*n* = 16), clinical relevance/core symptom (*n* = 8), improvement of care (*n* = 3), and supported by meta-analysis or research evidence (*n* = 3).

For the assessment of the 29 primary outcomes, 34 outcome measurement instruments were applied. It is striking that, except for one, all outcomes were measured by patient-reported outcome measures (PROMs). The use of these instruments was justified in the studies by their validity, reliability, sensitivity, or their common use.

However, even when approval studies embraced a similar primary outcome, they did not necessarily measure it using the same instruments. For example, quality of life was assessed in three approval studies^41,44,45^ using three different outcome measurement instruments: the Quality of Life Questionnaire for Patients on Long-Term Medication (QoLMed)^45^, the WHO Well-Being Index-5 (WHO 5)^41^, and the Short Form Health Survey- 12 item version (SF-12)^44^. While the QoLMed and SF-12 measure broader aspects of quality of life, the WHO-5 specifically focuses on psychological well-being, a key and specific component of quality of life. Primary outcomes were commonly measured directly at the end of the intervention. Drop-outs before the time of post-intervention measurement varied considerably across approval studies, with drop-out rates ranging from 4% to 53.3% (mean 21.7%; median 22%) in intervention groups and from 0% to 47% (mean 11.8%; median 8.6%) in control groups. In most studies, the drop-out rate before the post-intervention measurement was higher in the intervention than in the control group. It should also be noted that eight studies did not conduct a follow-up measurement^36,39–42,45,48,49^.

All approval studies found a statistically significant between-group effect in terms of primary outcome measurement. Approval studies that reported effect sizes according to Cohen’s d disclosed predominantly significant medium or large between-group effects for primary outcomes. The largest effect size for a primary outcome in terms of Cohen’s d was found for the improvement of insomnia severity (*d* = 1.79; *p* < 0.001)^43^, while the smallest was reported for the reduction of alcohol consumption, as measured by the Quantity/Frequency Index (QFI) (*d* = 0.34; *p* < 0.001)^36^.

Several other studies reported effects as relative or absolute changes, such as weight loss in kg or changes in instrument scores. In these cases, we could not assess effect sizes if study authors did not classify them. For example, without knowledge of the literature on obesity, it is not possible to tell whether a weight loss of 2.9 kg over the intervention period would constitute a small, medium, or large effect.

In addition to primary outcomes, the 23 included studies also examined a total of 120 secondary outcomes and 25 other outcomes.

Of the 120 secondary outcomes examined, 95 were assigned to the “medical benefit” outcome domain, specifically to the subcategories “improvement of health” (79), “improvement of quality of life” (15), and “prolongation of survival” (1). Further 12 secondary outcomes were assigned to the “patient-relevant improvements of structures and processes” outcome domain, specifically to the subcategories “adherence” (2), “patient safety” (2), “patient autonomy” (3), “health literacy“ (1), “coping with illness-related difficulties in everyday life” (3) and “reduction of therapy-related expenses and burdens for patients and their relatives” (1). The remaining 13 secondary outcomes were categorized as “other” outcomes, for example, “acceptance of diabetes” or “working ability”. For the measurement of the 120 secondary outcomes, 154 outcome measurement instruments were applied. Some instruments, such as the SF-12, were counted as two measurement instruments in cases where, for example, results for mental and physical health were analysed separately. The 25 other outcomes were measured by 29 outcome measurement instruments; in one additional case, the outcome measurement instrument was not specified. Three of these 25 other outcomes were not measured by questionnaires but by freely developed items and free text^39^. For 68 secondary outcomes and three other outcomes, a statistically significant between-group effect was shown. Of the 25 other outcomes, four outcomes were only measured for the intervention group, and 17 other outcomes were not included in the main analysis, meaning significant between-group effect at post-assessment could not be assessed.

### Risk of bias assessment

**Fig. 2:**
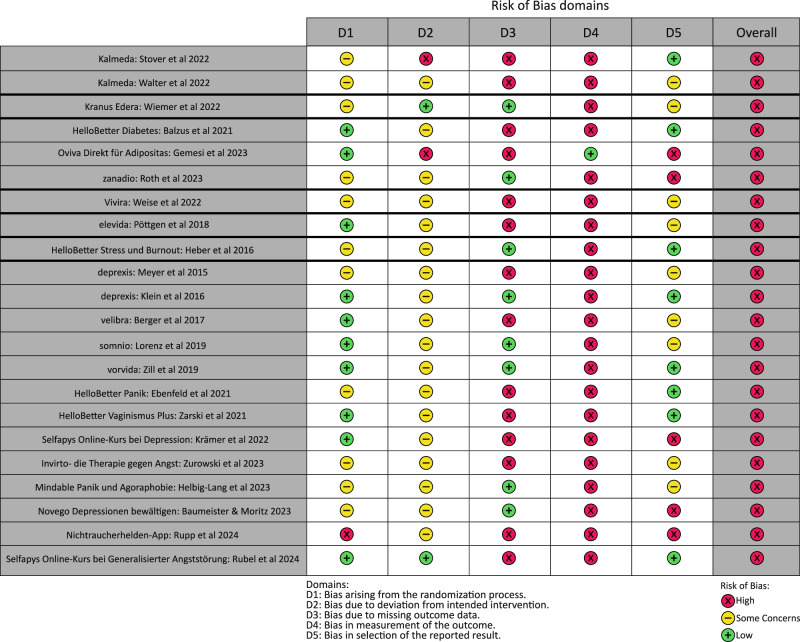
Results of the RoB2 assessment for 22 randomized parallel-group trials (ordered by DiGA category, then chronologically). The figure presents the RoB2 judgments for five bias domains and the overall risk of bias for each of the 22 included randomized parallel-group DiGA approval studies. Risk levels are color-coded as follows: green (low risk), yellow (some concerns), red (high risk). Figure 2 shows the studies ordered by DiGA category from the DiGA directory, as in Tables 1 and 2, then according to the chronological appearance of the study.

**Fig. 3:**
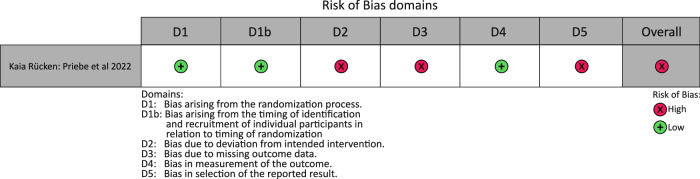
Results of the RoB2 assessment for cluster-randomized parallel-group trials. The figure presents the RoB2 judgments for five bias domains and the overall risk of bias for the included cluster-randomized parallel-group DiGA approval study. Risk levels are color-coded as follows: green (low risk), yellow (some concerns), red (high risk).

The RoB was assessed for all 23 included studies. All studies were evaluated to have an overall high RoB. The RoB results are presented for the 22 randomized parallel-group trials in Fig. 2 and for the cluster-randomized parallel-group trial in Fig. 3^34^. Risks relating to specific domains are presented below. The detailed RoB2 assessment is available in the Supplementary Information.

Regarding bias arising from the randomization process, the RoB was rated as low for 10 studies. However, some concerns were identified in 11 trials, and one study was assessed as having a high RoB^40^. Specifically, 19 trials used adequate randomization methods to generate the allocation sequence, and 11 studies appropriately concealed the allocation. Three studies did not clearly report randomization^19,38,48^ and 10 studies did not adequately describe allocation concealment methods. One study failed to adequately conceal allocation^40^.

With regard to bias due to deviations from the intended interventions, the RoB was rated as low for two studies^41,45^ and with some concerns for 18 studies. Two studies were assessed as having a high RoB^18,47^. A high RoB was primarily attributed to participants being aware of the intervention or to inadequate reporting of the likely influence of important, non-protocol interventions and the balance of these factors across study groups.

In terms of bias due to missing outcome data, the RoB was assessed as low for eight studies^16,36,39,42,43,45,48,51^, with the other 14 studies assessed as having a high RoB. High RoB was typically due to drop-out rates exceeding 5%, combined with a lack of analyses to provide evidence that the results were not biased by missing outcome data.

Regarding the bias in measurement of the outcome, the RoB was assessed as high for 21 studies. This was due to the predominant use of PROMs, which implies that outcomes may have been influenced by awareness of the intervention received. For one study^47^, the RoB was assessed as low because the primary outcome was measured objectively by study personnel during visits through various clinical parameters such as body weight, height, body composition, and BMI, with only the secondary outcome—quality of life—being patient-reported.

Concerning bias in the selection of reported results, the RoB was assessed as low for eight^16,18,35–37,41,46,51^ out of 22 studies. Low bias stemmed from all results of analyses and outcome measurements being reported, and analyses being conducted according to a pre-specified analysis plan outlined in the study protocol^22–25,27--30^. Some concerns persisted for one study^39^ for which a study protocol was available^32^, but discrepancies between the protocol and measured outcomes were identified. The RoB was also rated as some concern for eight further studies, for which results of all analyses and outcome measurements were reported, but no study protocol could be identified^17,19,38,43–45,49,50^. Five studies were assessed as having a high RoB due to selective reporting of outcome measurements, i.e., not reporting results for all measured outcomes, and/or analyses of the data^33,40,42,47,48^.

In addition to the parallel-group trials, the sole cluster-randomized parallel-group trial included in this review, published by Priebe et al.^34^, was assessed separately and found to have an overall high RoB (Fig. 3). Methodological flaws were identified in the three RoB domains bias due to deviations from the intended interventions, bias due to missing outcome data, and bias in selection of the reported results.

**Fig. 4:**
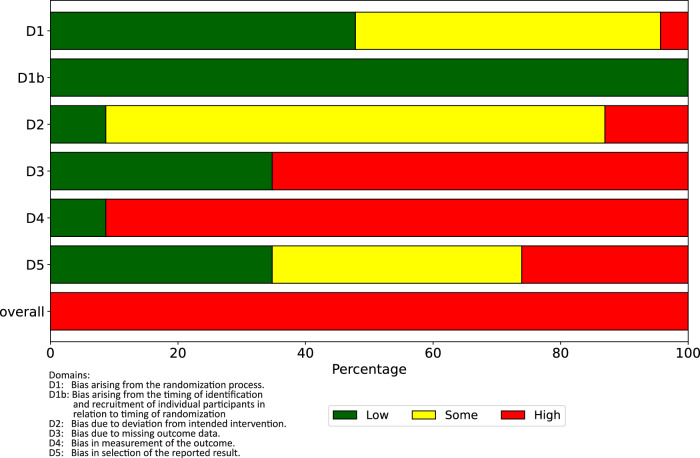
Overview of RoB across all included studies and domains. The figure displays the percentage distribution of RoB2 ratings (low risk, some concerns, high risk) across the five bias domains and overall for all 23 included studies.

In Fig. 4, the results of the RoB assessment across all RoB domains are visualized for the 23 included studies. As the figure shows, across all studies, the main sources of high RoB are in the domains bias due to missing outcome data and bias in measurement of the outcome. The domain bias due to deviation from intended interventions is a source of some concerns, while the randomization process was appropriately conducted in the majority of studies.

## Discussion

The objective of this systematic review of DiGA approval studies was two-fold: first, to give an overview of the study characteristics, measurement instruments, outcomes, and positive healthcare effects relating to these tools; and, second, to assess the RoB of studies submitted by manufacturers to the BfArM for permanent approval for their DiGAs.

In total, 23 approval studies for 21 DiGAs published as of March 15, 2024, were included in this review.

The results revealed substantial differences among the studies. This was first of all evident with regard to intervention design and study characteristics, including sample sizes, drop-out rates, measurement times, and intervention durations. These variations between interventions reflected the flexibility granted to manufacturers in designing interventions within the German DiGA approval process. Another source of variability between interventions was in the choice and measurement of primary, secondary, and additional outcomes. Despite this, an overview of the outcomes revealed that all approval studies reported statistically significant and predominantly medium to large between-group effects for their primary outcomes. In contrast, the results for secondary outcomes were more variable.

The RoB showed that the overall RoB was high for all approval studies, albeit with variation across different domains.

Thus, the main conclusions of this systematic review are that DiGA approval studies are difficult to compare and that evidence provided for the positive healthcare effects of DiGAs should be critically evaluated, as results are prone to bias.

An overview of measured outcomes was not conducted in previous reviews of DiGA approval studies^7,8^. Thus, no comparison of findings is possible. A discussion of the effects reported by approval studies in comparison to previous research is also impossible; first, due to the wide range of outcomes examined by approval studies, and second, because of the novelty and international singularity of DiGA. The comparability with effects found for other mobile health applications is questionable.

In assessing the RoB, our systematic review added to the literature by evaluating several approval studies that had not been included in former reviews. The overall RoB assessment we present is consistent with two former systematic reviews that examined several DiGA approval studies published before 2022^7,8^. Both reviews also found a high overall RoB across all examined DiGA approval studies, with a varying RoB across different domains. Comparing our findings on underlying methodological issues in the additional studies with the findings of these two previous studies, it can be concluded that the RoB in newer approval studies does not systematically differ from that of earlier studies. Neither the domain-specific RoB assessments nor the overall RoB ratings showed systematic improvement over time. The lack of systematic improvement over time may in part be explained by the fact that the methodological requirements outlined in the DiGA fast-track guidelines have remained largely unchanged since their introduction.

Although not specifically required by DiGA guidelines^2^, all approval studies were conducted using a (cluster-)randomized controlled trial design, considered the gold standard for clinical evidence.

Across approval studies, there were substantial differences in sample sizes and drop-out rates, with very small and very large examples of each. This variation may affect the interpretability and comparability of the study results. However, as emphasized in the DiGA fast-track guidelines, both sample sizes and drop-out rates should only be evaluated in comparison with similar interventions^2^. Thus, a small sample size or a high drop-out rate is not necessarily unfavorable. In several approval studies, the observed drop-out rates were higher in the intervention than in the control group. Assessing the causes and implications of this phenomenon is more difficult and does not allow for definitive conclusions.

Possible explanations for drop-outs in the intervention group might be that the app does not meet expectations, for example, with regard to an observed positive healthcare effect, or might not align with participants´ daily routines. However, the literature suggests that different factors are underlying high drop-out rates in digital interventions. A meta-analysis by Torous et al.^52^ dealing with drop-out rates in clinical trials of smartphone apps for depressive symptoms found no significant differences in drop-out rates between intervention and waitlist control groups. Torous et al. identify in-app mood monitoring and the provision of human feedback as key factors in reducing attrition rates, while features of intervention design, such as including waitlist or placebo controls, clinical vs. non-clinical populations, and therapeutic approaches to depression had no effect on drop-out rates.

Beyond clinical trials, high drop-out rates in the intervention group also indicate a challenge to translate DiGA into real-world healthcare settings, particularly regarding adherence and utilization following prescription. Reasons for low adherence may include insufficient user support, lack of individualization, usability issues, and poor integration into routine care^53,54^. Ultimately, low utilization may negatively impact the overall effectiveness of DiGAs.

Some interventions had a relatively short duration of three months or less, and in some cases, no follow-up assessment was conducted. This is in line with the DiGA fast-track guidelines from the BfArM; however, such a short intervention duration and a lack of follow-up are questionable study design elements. In practice, DiGAs are often prescribed multiple times or for a longer duration, as they mostly address chronic diseases. Thus, it may be appropriate to include obligatory analysis of long-term positive healthcare effects of DiGAs as part of the approval process.

Overall, the outlined divergence of approval studies is likely the result of the far-from-strict BfArM DiGA fast-track guidelines, which leave manufacturers a large scope for study design, especially when compared to more strictly regulated processes such as the AMNOG procedure. This leads to low comparability both between DiGA approval studies in general and between DiGAs designed for the same indication.

The reporting of outcomes and the outcome measurement instruments applied by approval studies revealed further marked differences. This is not only attributable to the variety of indications addressed, but also to the fact that approval studies employed different outcomes and measurement instruments even for similar indications. Finally, the diversity of outcomes and measurement instruments makes it difficult to compare approval studies and their reported positive healthcare effects. One way to enhance comparability would be to mandate the use of core outcome sets (COS)^55^ in approval studies. For example, for the indications depression and anxiety, a COS is available^56^, which comprises four general treatment outcomes: symptom burden, functioning, disease progression, and treatment sustainability, as well as potential side effects of treatments. This COS could have been applied in the approval studies of DiGA for depression and/or anxiety. However, specific COS for digital interventions have yet to be developed and validated.

Registers might also play a valuable role in enabling a comparable, cross-DiGA measurement of predefined outcomes- ideally based on standardized outcome sets. A recent publication by Albrecht et al.^57^ illustrates this approach by combining overarching and indication-specific outcomes within the DiGAReal registry for rheumatology patients.

As outlined above, the overall RoB for all approval studies was assessed as high, with different underlying causes. One cause was the suboptimal reporting quality of the included studies. Missing information or imprecise wording sometimes means that a RoB cannot be ruled out; for example, if it is not clear whether an allocation sequence in the randomization process was concealed or not, the possibility that it was not must be considered. Related to this, study protocols were often not publicly available, meaning it was not possible to assess whether data analyses were carried out according to a pre-specified analysis plan. A second cause of poor RoB ratings was the use of inadequate statistical models to deal with missing data due to drop-outs, reflected in the high RoB scores for missing outcome data. Another cause of high RoB was the frequent use of PROMs, such as those assessing improvements in quality of life, combined with the absence of blinding of outcome assessors.

While poor RoB ratings due to suboptimal reporting quality, missing study protocols, and inadequate statistical analyses can be easily avoided by better adherence to the CONSORT reporting guidelines—as already suggested in the DiGA fast track guidelines—or through the publication of study protocols, the use of PROMs is a more challenging issue.

The use of PROMs to assess DiGA outcomes is an obvious and necessary choice, as the intended effects of DiGA—such as improved quality of life—are often difficult or impossible to measure objectively. Additionally, DiGA interventions are conducted outside of clinical settings and are predominantly self-guided. Consequently, employing a waitlist study design seems reasonable, as the use of “sham” apps for control groups would be difficult to implement and likely methodologically inadequate^7^. Thus, overcoming the bias arising from participants’ awareness of their treatment allocation remains challenging. Nevertheless, it may be worth discussing whether the RoB2 tool´s classification of PROM usage—an essential part of patient-centered care- as a potential source of bias is always justified or appropriate.

These findings must be interpreted in light of certain strengths and limitations of the present review.

Our review provides an overview of study characteristics, measurements, outcomes, and the effects reported by DiGA approval studies, as well as an assessment of their RoB. Unlike previous reviews^7,8^, it also processes the reported effects, representing significant added value.

The insights gained from this review are valuable for both scientific and practical purposes. For the scientific audience, a comprehensive overview of the positive healthcare effects of DiGA and the associated RoB in the supporting evidence fills a knowledge gap. In practice, this information can offer context and guidance for DiGA prescribers. Also, by highlighting methodological gaps and inconsistencies, it establishes a framework to aid manufacturers and stakeholders in improving the design of future approval studies and the DiGA approval process, particularly a refinement of the fast-track guidelines.

This review also has limitations. We only included DiGA approval studies that demonstrated a positive healthcare effect on the basis of which DiGA received permanent approval. Studies that failed to demonstrate such an effect and as a result a DiGA did not receive permanent approval could not be considered, as these studies are not publicly available for further analysis. The BfArM provides only very limited information on the reasons why permanent approval was not granted to provisionally approved and withdrawn DiGA (see introduction). Moreover, it is not known how many applications for direct permanent approval were rejected by the BfArM.

Identifying all relevant approval studies was challenging. Although the search strategy was adjusted to address this issue—by incorporating various search methods, such as the DiGA directory, websites, MEDLINE, and a manual search via Google Scholar—it is possible that not all published approval studies for the 33 DiGAs permanently approved as of March 15, 2024, were identified and included in this review. Retrieval of DiGA approval studies could be facilitated by requiring manufacturers to provide a link to the relevant study in the DiGA directory. Regarding data extraction, it must be noted that not all required information could be extracted from the studies due to the sometimes poor reporting quality. Ambiguities could potentially have been resolved through communication with the study authors; however, this is not in line with the principles of the RoB2.

However, the RoB 2 guidelines recommend contacting study authors to request study protocols that are not publicly accessible^58^. Yet, in the DiGA approval process, the publication of approval studies and study registration is mandatory. It would therefore be consistent and appropriate if corresponding study protocols were also published as part of this regulatory transparency. Consequently, we refrained from contacting study authors to request unpublished protocols. In cases where no protocol was publicly available, we documented this in the RoB assessment and the RoB rating in the section was “no information”. This applied to 11 included DiGA studies. To enhance the clarity of evidence for the positive healthcare effects of DiGAs, it could be beneficial for the BfArM to mandate adherence to reporting guidelines for future approval studies.

Furthermore, the categorization of primary and secondary outcomes—as either “medical benefit” or “patient-relevant improvement of structures and processes”—had to be conducted to the best of our knowledge, due to the lack of clear definitions in the DiGA fast-track guidelines.

In several studies, the statistical significance or effect sizes were not clearly specified for reported outcomes. Consequently, data extraction required the interpretation of the available information to the best of our knowledge. Effect sizes were reported in Table 2 as indicated by the authors of the approval studies. This is because the studies used a wide range of outcomes and a variety of measurement instruments. Independent interpretation and contextualization of the effect sizes would require detailed knowledge of all these instruments and related literature, which was not feasible within the scope of this review.

The quality assessment was conducted across outcomes, taking into consideration the diversity of outcomes in the studies and the lack of comparability between them. Further analyses, such as meta-analysis, could not be conducted due to the diversity of outcomes and conditions applied in the approval studies. Thus, we were limited to a narrative presentation of the results.

The findings of our systematic review have several implications for practice and future research. The RoB assessment, in particular, clearly suggests that the evidence presented for the positive healthcare effects of DiGAs in approval studies should be considered with caution. However, a review of the DiGA fast-track guidelines reveals that the included studies do indeed fulfill the criteria for permanent approval, the granting of which is a discretionary decision made by the BfArM.

Making the permanent approval of DiGAs a discretionary decision may be considered reasonable at first glance, taking into account the diversity of DiGA interventions. However, given the divergence between scientific standards for high-quality studies and the approval criteria for DiGAs established by BfArM guidelines, a discussion on how to narrow this gap is imperative.

As the BfArM continues to grant permanent approval for DiGAs, improving the methodological quality of approval studies is important. Improving the methodological quality of DiGA studies would help to ensure trust in and the quality of DiGAs, which is crucial given the planned approval of certified DiGAs as medical devices of risk class II.

Aiming to improve the methodological quality of DiGA approval studies, the results of this systematic review imply several possible measures that could be adopted by the BfArM and manufacturers.

First, adaptations to the criteria for permanent approval of DiGA could be key to improving the quality of manufacturers’ studies. As methodological problems of early approval studies do not differ from those of later ones, a connection to the fast-track guidelines appears evident.

Thus, to address poor reporting quality, the prospective publication of study protocols and reporting according to CONSORT guidelines should be mandatory. To improve comparability between approval studies and the effectiveness of DiGAs, the BfArM should demand clear rationales for outcome selection and recommend adherence to COS. Also, a minimum intervention duration and the conduct of a follow-up should be recommended, as DiGAs often address chronic diseases and are thus prescribed multiple times and for more than 90 days at a time.

To address the RoB resulting from the inadequate use of statistical models for dealing with missing data, e.g., last observation carried forward (LOCF), the BfArM could provide details of recommended models in its guidelines. These criteria should be communicated transparently to DiGA manufacturers via the DiGA fast-track guidelines to ensure clarity and consistency in expectations. The consequences of inadequate reporting quality and methodological flaws should also be clearly communicated.

Second, incorporating real-world evidence as a mandatory component of the approval process could help strengthen the evidence base by enabling the monitoring of DiGA performance. The introduction of an application-accompanying performance measurement (in German “anwendungsbegleitende Erfolgsmessung”, AbEM) for DiGA in Germany from 2026^59^ might be a valuable step in this direction.

Third, the BfArM should increase the transparency of the approval process. As permanent approval of a DiGA is a discretionary decision by the BfArM, the criteria used to evaluate the quality of DiGA approval studies must be publicly available. It would also be reasonable for the BfArM to apply standardized instruments, such as the RoB tool, for evaluating approval studies as part of its decision-making process. To further enhance transparency, the BfArM should also publish more detailed information on rejected applications for permanent approval and the rationales behind these decisions. In doing so, the BfArM could enhance trust in its decisions and provide an example of best practice for manufacturers designing future DiGA approval studies. Relatedly, the BfArM should also be transparent about how it handles violations of, or exceptions to, the 12-month publication deadline for approval studies of permanently approved DiGAs. According to our research, as of June 02, 2025, the 12-month deadline had expired for five permanently approved DiGAs. Clear communication on whether and how such delays affect the approval status would strengthen accountability and provide important guidance for manufacturers and international stakeholders alike.

Beyond the recommendations for improving the approval process in Germany, this systematic review offers important insights and valuable lessons for the international context. As Germany has been a pioneer in establishing reimbursable digital health applications, other countries, including France, Belgium, and Austria have already modeled their processes after the German fast-track procedure^60^. Other countries may follow in the future. Therefore, an important takeaway from this review is the importance of the early implementation of adequate mechanisms in the approval process to systematically identify methodological weaknesses in approval studies for DiGAs. Ensuring transparency around these mechanisms could enhance international comparability and foster the adoption of best practices globally.

Taken together, the findings of this systematic review highlight that DiGA approval studies exhibit potential for methodological improvement and should be closely monitored. In an update of this systematic review, we will review approval studies published after March 15, 2024. While the focus of the present review was on outcomes measured post-intervention, the focus of the next review will be on follow-up measurements and evidence for the long-term positive healthcare effects of DiGAs.

## Methods

The systematic review was registered prospectively with PROSPERO at https://www.crd.york.ac.uk/prospero/ (CRD42023460497) and adheres to the PRISMA (Preferred Reporting Items for Systematic Reviews and Meta-Analyses) guidelines to ensure comprehensive and transparent reporting^61^. The PRISMA checklist is available in the Supplementary Information.

### Study inclusion and exclusion

This systematic review includes all studies that were submitted by DiGA manufacturers to the BfArM to present evidence for positive health care effects of the DiGA and that are (a) published in either German or English and (b) publicly available as a full text as of March 15, 2024, in any format (study reports, journal articles). In addition, we included all publicly available corresponding study protocols.

### Search strategy and selection process

According to the DiGA directory (https://diga.bfarm.de), a total of 33 DiGAs were granted permanent approval by the BfArM as of March 15, 2024. Hence, a search for approval studies of the 33 DiGA was conducted. As a first step, the DiGA directory was consulted. As the directory comprises a registry where manufacturers should identify the approval studies relevant to their DiGA, we expected to retrieve all approval studies from this source. However, approval studies were cited for less than half of the permanently approved DiGAs. In most cases, the listing was either left blank or contained references to the manufacturers’ websites. Thus, the search strategy was readjusted by including additional sources. If the approval study was not cited in the DiGA directory, the studies were searched for first on the manufacturers’ websites. If the study could not be identified from the website, searches were conducted in MEDLINE using PubMed and manually in Google Scholar using the study registration number. The search for approval studies was repeated monthly, beginning on 15 September 2023 and concluding on March 15, 2024, to identify newly published approval studies.

To ensure that identified studies were the relevant approval study for a given DiGA, details were checked against available information from the DiGA directory, study registries, and manufacturers’ websites on study characteristics such as sample size, period of intervention, and trial registration numbers. In addition, study protocols corresponding to the approval studies were searched for via the German Clinical Trials Register (DRKS) and clinicaltrials.gov, manufacturers’ websites, and manually on Google Scholar using the study registration number published in the DiGA directory^5^. As all approval studies were to be included in this review, no title-abstract-screening and no full-text-screening was performed. Hence, a discussion and an agreement between the reviewers on the selection of eligible studies was not conducted.

### Data extraction

After the identification of approval studies and corresponding study protocols, data extraction was conducted by one researcher (KS) and verified for accuracy and completeness by a second (MS). Any disagreements between the reviewers were resolved through discussion or with the assistance of a third reviewer (SD).

Data extraction was performed using a data extraction sheet in tabular form, developed by two reviewers (KS, MS) and tested on two randomly selected included studies to ensure that all relevant information of the study was extracted.

The data extraction sheet comprised categories based on the population, intervention, control, outcome, and study design (PICOS) scheme, as well as complementary information deemed relevant. The categories were (1) citation details, (2) study design, (3) recruitment and inclusion/exclusion criteria, (4) population characteristics and sample size, (5) country of intervention, (6) the allocation of the study participants to the intervention and control group(s), (7) intervention or treatment applied to intervention and control group(s), (8) drop-outs, (9) methods of analysis, (10) outcomes, (11) outcome measurement instruments, (12) measurement times, (13) effect of intervention, (14) effect size.

Information on the rationale for outcome selection was also collected from each study, with a focus on whether core outcome sets were applied, as proposed by Williamson et al.^55^ These rationales were categorized according to a system developed by KS, MS, and SD as part of the review process. Overall, a distinction was made between implicit and explicit rationales for outcome selection.

A rationale was considered implicit if

1. The condition or symptom being measured is very common, making it essential to focus on reducing its impact (high prevalence of condition).
2. The outcome directly reflects the core symptoms or conditions being treated, and reducing these symptoms is a key goal of the intervention (clinical relevance/core symptom).
3. The outcome was chosen because provision of healthcare for treatment of this condition or symptom could be improved, including better access, more efficient treatment, or overcoming barriers in traditional care (improvement of care).
4. The outcome was selected because the condition or symptom being measured has a substantial negative effect on patients’ daily functioning and overall well-being (high burden or impact on quality of life).
5. The outcome was selected based on strong empirical support from previous studies, systematic reviews, or meta-analyses that have demonstrated its relevance in evaluating treatment across multiple research settings (supported by meta-analysis or research evidence).

In contrast, a rationale was considered explicit if either the relevance of the outcome was determined in a structured manner—such as by conducting a Delphi survey or focus group with patients or experts—or the outcome was chosen with reference to a core outcome set.

With regard to treatment effects, this review focused on the reporting of primary outcomes as identified by study authors, measured at post-intervention. This is because the proof of a long-term positive healthcare effect for a primary outcome is not mandatory for the permanent approval of a DiGA by the BfArM. Consequently, follow-up measurements are not obligatory and are likely not included in all studies.

To assess effect sizes, we took classifications from the approval studies when available; otherwise, we assessed effect sizes according to standard statistical literature. If study authors reported relative improvements but did not specify an effect size, no categorization was made, as expert knowledge would be necessary to make such a determination.

### Risk of bias assessments

As of March 15, 2024, the studies submitted to the BfArM were either randomized controlled or cluster-randomized controlled trials. Thus, for the RoB assessment, the RoB2 tool for randomized controlled trials^15^ (August 22, 2019 version) and the adapted RoB2 tool for cluster-randomized controlled trials (March 18, 2020 version) were applied^15,58,62^.

The Cochrane RoB2 tool is structured into five domains of bias that focus on different aspects of trial design, conduct, and reporting. These five domains are (1) RoB arising from the randomization process, (2) RoB due to deviation from intended intervention, (3) RoB due to missing outcome data, (4) RoB in measurement of the outcome, and (5) RoB in selection of the reported result. For each domain, the RoB2 provides a fixed series of questions to collect information on the possible RoB, and an algorithm with signaling questions to generate the RoB assessment for each domain; an overall RoB is then determined at the study level. A rating out of three possible outcomes is given for each domain and the study overall: “low,” “some concerns,” or “high.”

In the RoB assessment, the effect of assignment to intervention was of particular interest. The RoB2 was used to assess the risk associated with the outcome(s), outcome measure(s), and timepoint(s)^58^. We anticipated that outcomes in approval studies would be heterogeneous because of variations in scope, target groups, addressed conditions, and overall aims of DiGAs. Thus, for consistency, the RoB assessment was conducted per study instead of per outcome.

Previous RoB assessments have been conducted for some approval studies as part of previously published systematic reviews^7,8^; however, due to differences in some assessed domains, these studies were included in the assessment here as well.

The RoB in the identified studies was assessed independently by two researchers (KS, SD), using the RoB2 Excel tool provided on the riskofbiasinfo.org website. As the literature suggests that training on the use of the tool is necessary to correctly apply the tool and improve inter-rater agreement^63^, three studies were initially selected for calibration in order to develop a shared understanding of the RoB2 tool. Results of the assessment were discussed by the two researchers, and consensus was reached. Disagreements between the two researchers were observed in domain D3 (“bias due to missing outcome data”), due to the complexity of the statistical analysis models used, and in domain D5 (“bias in selection of the reported result)”, where the assessment requires a particularly high degree of interpretation regarding the quality and completeness of reporting.

## Supplementary information

Supplementary information

Supplementary Data 1

Supplementary Data 2

Supplementary Data 3

## Data Availability

All data extracted and analyzed in this systematic review are based on publicly available sources as cited in the manuscript. The complete data extraction table is available as Supplementary Data 1. The detailed RoB2 assessments are included in the Supplementary Information file as Supplementary Table 2.

## Abbreviations

AbEM: Application-accompanying performance measurement (in German “anwendungsbegleitende Erfolgsmessung”)
AMNOG: German Medicines Market Reorganisation Act (in German “Arzneimittelmarktneuordnungsgesetz”)
BfArM: Federal Institute for Drugs and Medical Devices (in German “Bundesinstitut für Arzneimittel und Medizinprodukte”)
COS: Core outcome sets
DiGA: Reimbursable digital health application in Germany (in German “Digitale Gesundheitsanwendungen”)
DigiG: Digital Act (in German “Digital-Gesetz”)
DVG: Digital Healthcare Act (in German “Digitale-Versorgungs-Gesetz”)
DRKS: German Clinical Trials Register (in German “Deutsches Register für Klinische Studien”)
MDD: Medical Device Directive
G-BA: Federal Joint Comittee (in German “Gemeinsamer Bundesausschuss“)
MDR: Medical Device Regulation
PROM: patient-reported outcome measure
psVV: Patient-relevant improvement in structures and processes (in German “patientenrelevante Struktur- und Verfahrensverbesserung”)
RoB: Risk of Bias
RoB2: Risk of Bias Tool Version 2

## Code availability

No code was used for data pre-processing or statistical analysis. Figures 2 and 3 were created using Inkscape v1.3.2. Visualization and layout are based on the open-source tool robvis (https://github.com/mcguinlu/robvis), with minor modifications for visualization purposes. Figure 4 was generated using Python v3.11.10. All adapted code for figure generation is available from the corresponding author upon reasonable request.
