# Supplementary material for: Healthcare effects and evidence robustness of reimbursable digital health applications in Germany: a systematic review
Source: NPJ Digit Med. 2025 Aug 1;8:495. doi: 10.1038/s41746-025-01879-6 (PMC12317029; doi:10.1038/s41746-025-01879-6)
Supplement: Supplementary file 1 — Supplementary information [file 41746_2025_1879_MOESM1_ESM.pdf]

1. PRISMA Checklist

Completed PRISMA checklist documenting compliance with reporting standards for systematic reviews.

Section and Topic	Item #	Checklist item	Location where item is reported
**TITLE**			
Title	1	Identify the report as a systematic review.	Title page
**ABSTRACT**			
Abstract	2	See the PRISMA 2020 for Abstracts checklist.	The PRISMA 2020 for Abstracts checklist was followed to the extent permitted by the journal's word count restrictions.
**INTRODUCTION**			
Rationale	3	Describe the rationale for the review in the context of existing knowledge.	Introduction
Objectives	4	Provide an explicit statement of the objective(s) or question(s) the review addresses.	Introduction
**METHODS**			
Eligibility criteria	5	Specify the inclusion and exclusion criteria for the review and how studies were grouped for the syntheses.	Methods, Study inclusion
Information sources	6	Specify all databases, registers, websites, organisations, reference lists and other sources searched or consulted to identify studies. Specify the date when each source was last searched or consulted.	Methods, Search strategy
Search strategy	7	Present the full search strategies for all databases, registers and websites, including any filters and limits used.	Methods, Search strategy
Selection process	8	Specify the methods used to decide whether a study met the inclusion criteria of the review, including how many reviewers screened each record and each report retrieved, whether they worked independently, and if applicable, details of automation tools used in the process.	Methods, Search strategy
Data collection process	9	Specify the methods used to collect data from reports, including how many reviewers collected data from each report, whether they worked independently, any processes for obtaining or confirming data from study investigators, and if applicable, details of automation tools used in the process.	Methods, Data extraction
Data items	10a	List and define all outcomes for which data were sought. Specify whether all results that were compatible with each outcome domain in each study were sought (e.g. for all measures, time points, analyses), and if not, the methods used to decide which results to collect.	Methods, Data extraction
	10b	List and define all other variables for which data were sought (e.g. participant and intervention characteristics, funding sources). Describe any assumptions made about any missing or unclear information.	Methods, Data extraction
Study risk of bias	11	Specify the methods used to assess risk of bias in the included studies, including details of the tool(s) used, how many reviewers	Methods, Risk of

Section and Topic	Item #	Checklist item	Location where item is reported
assessment		assessed each study and whether they worked independently, and if applicable, details of automation tools used in the process.	bias assessment
Effect measures	12	Specify for each outcome the effect measure(s) (e.g. risk ratio, mean difference) used in the synthesis or presentation of results.	Table 2
Synthesis methods	13a	Describe the processes used to decide which studies were eligible for each synthesis (e.g. tabulating the study intervention characteristics and comparing against the planned groups for each synthesis (item #5)).	NA, as no quantitative synthesis was conducted
	13b	Describe any methods required to prepare the data for presentation or synthesis, such as handling of missing summary statistics, or data conversions.	NA
	13c	Describe any methods used to tabulate or visually display results of individual studies and syntheses.	NA, all identified approval studies were included.
	13d	Describe any methods used to synthesize results and provide a rationale for the choice(s). If meta-analysis was performed, describe the model(s), method(s) to identify the presence and extent of statistical heterogeneity, and software package(s) used.	As no meta-analysis was conducted, results were synthesized narratively and presented in structured tables (1-2) plus appendices.
	13e	Describe any methods used to explore possible causes of heterogeneity among study results (e.g. subgroup analysis, meta-regression).	NA
	13f	Describe any sensitivity analyses conducted to assess robustness of the synthesized results.	NA
Reporting bias assessment	14	Describe any methods used to assess risk of bias due to missing results in a synthesis (arising from reporting biases).	NA
Certainty assessment	15	Describe any methods used to assess certainty (or confidence) in the body of evidence for an outcome.	NA
**RESULTS**			
Study selection	16a	Describe the results of the search and selection process, from the number of records identified in the search to the number of studies included in the review, ideally using a flow diagram.	Results of the search process
	16b	Cite studies that might appear to meet the inclusion criteria, but which were excluded, and explain why they were excluded.	Results of the search process
Study characteristics	17	Cite each included study and present its characteristics.	Table 1+ Table 2
Risk of bias in studies	18	Present assessments of risk of bias for each included study.	Results of RoB assessment

Section and Topic	Item #	Checklist item	Location where item is reported
Results of individual studies	19	For all outcomes, present, for each study: (a) summary statistics for each group (where appropriate) and (b) an effect estimate and its precision (e.g. confidence/credible interval), ideally using structured tables or plots.	Table 2, Supplementary Data 1-3
Results of syntheses	20a	For each synthesis, briefly summarise the characteristics and risk of bias among contributing studies.	Figure 2
	20b	Present results of all statistical syntheses conducted. If meta-analysis was done, present for each the summary estimate and its precision (e.g. confidence/credible interval) and measures of statistical heterogeneity. If comparing groups, describe the direction of the effect.	NA
	20c	Present results of all investigations of possible causes of heterogeneity among study results.	NA
	20d	Present results of all sensitivity analyses conducted to assess the robustness of the synthesized results.	NA
Reporting biases	21	Present assessments of risk of bias due to missing results (arising from reporting biases) for each synthesis assessed.	NA
Certainty of evidence	22	Present assessments of certainty (or confidence) in the body of evidence for each outcome assessed.	NA
**DISCUSSION**			
Discussion	23a	Provide a general interpretation of the results in the context of other evidence.	Discussion
	23b	Discuss any limitations of the evidence included in the review.	Discussion
	23c	Discuss any limitations of the review processes used.	Discussion
	23d	Discuss implications of the results for practice, policy, and future research.	Discussion
**OTHER INFORMATION**			
Registration and protocol	24a	Provide registration information for the review, including register name and registration number, or state that the review was not registered.	Abstract
	24b	Indicate where the review protocol can be accessed, or state that a protocol was not prepared.	Abstract
	24c	Describe and explain any amendments to information provided at registration or in the protocol.	NA
Support	25	Describe sources of financial or non-financial support for the review, and the role of the funders or sponsors in the review.	Acknowledgements
Competing interests	26	Declare any competing interests of review authors.	Competing interests
Availability of data, code and other materials	27	Report which of the following are publicly available and where they can be found: template data collection forms; data extracted from included studies; data used for all analyses; analytic code; any other materials used in the review.	Supplementary Data 1-3

From: Page MJ, McKenzie JE, Bossuyt PM, Boutron I, Hoffmann TC, Mulrow CD, et al. The PRISMA 2020 statement: an updated guideline for reporting systematic reviews. BMJ 2021;372:n71. doi: 10.1136/bmj.n71

2. Detailed Risk of Bias 2 (RoB2) Assessments

Detailed domain-level RoB2 ratings for all 23 included DiGA approval studies. Judgments are listed for each of the five bias domains and the overall risk of bias. Part 1 contains the assessments for 22 randomized parallel-group trials. Part 2 presents the assessment of the single included cluster-randomized trial.

Part 1: RoB assessment of parallel RCTs

First author (publication year)	Klein et al 2016	Meyer et al 2015	Heber et al 2016	Berger et al 2017	Pöttgen et al 2018	Lorenz et al 2019	Zill et al 2019	Balzus et al 2021
DiGA	deprexis	deprexis	HelloBetter Stress & Burnout	velibra	elevida	somnio	vorvida	HelloBetter Diabetes und Depression
Outcome (time of measurement) that is being assessed for risk of bias								
1.1 Was the allocation sequence random?	Y	Y	Y	Y	Y	Y	Y	Y
Comments								
1.2 Was the allocation sequence concealed until participants were enrolled and assigned to interventions?	Y	NI	NI	Y	Y	Y	Y	PY
Comments								Automatic randomization, probably self-enrollment
1.3 Did baseline differences between intervention groups suggest a problem with the randomization process?	NI	N	NI	NI	NI	NI	N	N
Comments	No test for significance		?	No test for significance	No test for significance	No test for significance		
Risk of bias arising from the randomization process	low	Some concerns	Some concerns	low	low	low	low	low
2.1. Were participants aware of their assigned intervention during the trial?	Y	Y	Y	Y	Y	Y	Y	PY
Comments								Active control group.

First author (publication year)	Klein et al 2016	Meyer et al 2015	Heber et al 2016	Berger et al 2017	Pöttgen et al 2018	Lorenz et al 2019	Zill et al 2019	Balzus et al 2021
2.2. Were carers and people delivering the interventions aware of participants' assigned intervention during the trial?	Y	N	Y	N	N	N	N	N
Comments	Guided use of app for people with severe symptoms							
2.3. If Y/PY/NI to 2.1 or 2.2: Were there deviations from the intended intervention that arose because of the trial context?	NI	NI	NI	NI	NI	NI	NI	NI
Comments								
2.4 If Y/PY to 2.3: Were these deviations likely to have affected the outcome?	NA	NA	NA	NA	NA	NA	NA	NA
Comments								
2.5. If Y/PY/NI to 2.4: Were these deviations from intended intervention balanced between groups?	NA	NA	NA	NA	NA	NA	NA	NA
Comments								
2.6 Was an appropriate analysis used to estimate the effect of assignment to intervention?	Y	Y	Y	Y	Y	NI	Y	Y
Comments								
2.7 If N/PN/NI to 2.6: Was there potential for a substantial impact (on the result) of the failure to analyse participants in the group to which they were randomized?	NA	NA	NA	NA	NA	N	NA	NA
Comments								
Risk of bias due to deviations from the intended interventions	Some concerns	Some concerns	Some concerns	Some concerns	Some concerns	Some concerns	Some concerns	Some concerns
3.1 Were data for this outcome available for all, or nearly all, participants randomized?	N	N	N	N	N	Y	N	N
Comments								
3.2 If N/PN/NI to 3.1: Is there evidence that the result was not biased by missing outcome data?	PY	N	PY	N	N	NA	Y	PN

First author (publication year)	Klein et al 2016	Meyer et al 2015	Heber et al 2016	Berger et al 2017	Pöttgen et al 2018	Lorenz et al 2019	Zill et al 2019	Balzus et al 2021
Comments			Multiple imputation to correct for bias		Sensitivity analysis/multiple analysis not convincing			
3.3 If N/PN to 3.2: Could missingness in the outcome depend on its true value?	NA	PY	NA	Y	NI	NA	NA	NI
Comments								
3.4 If Y/PY/NI to 3.3: Is it likely that missingness in the outcome depended on its true value?	NA	PY	NA	NI	PY	NA	NA	NI
Comments								
Risk of bias due to missing outcome data	Low	High	Low	high	high	low	low	high
4.1 Was the method of measuring the outcome inappropriate?	N	N	N	N	N	N	N	N
Comments								
4.2 Could measurement or ascertainment of the outcome have	N	N	N	N	N	N	N	N

First author (publication year)	Klein et al 2016	Meyer et al 2015	Heber et al 2016	Berger et al 2017	Pöttgen et al 2018	Lorenz et al 2019	Zill et al 2019	Balzus et al 2021
differed between intervention groups?								
Comments								
4.3 If N/PN/NI to 4.1 and 4.2: Were outcome assessors aware of the intervention received by study participants?	PY	Y	Y	Y	Y	Y	Y	PY
Comments								
4.4 If Y/PY/NI to 4.3: Could assessment of the outcome have been influenced by knowledge of intervention received?	PY	Y	Y	Y	Y	Y	Y	PY
Comments								
4.5 If Y/PY/NI to 4.4: Is it likely that assessment of the outcome was influenced by knowledge of intervention received?	NI	NI	NI	NI	NI	NI	NI	NI
Comments								
Risk of bias in measurement of the outcome	high	high	high	high	high	high	high	high
5.1 Were the data that produced this result analysed in accordance with a pre-specified analysis plan that was finalized before unblinded outcome data were available for analysis?	Y	NI	Y	NI	NI	NI	Y	Y
Comments		No study protocol published, no information on analysis in study registry		No study protocol published, no information on analysis in study registry	No study protocol published, no information on analysis in study registry	No study protocol published, no information on analysis in study registry		
5.2 Is the numerical result being assessed likely to have been selected, on the basis of the results, from multiple eligible outcome measurements within the outcome domain?	N	N	N	N	N	N	N	N
Comments								

First author (publication year)	Klein et al 2016	Meyer et al 2015	Heber et al 2016	Berger et al 2017	Pöttgen et al 2018	Lorenz et al 2019	Zill et al 2019	Balzus et al 2021
5.3 Is the numerical result being assessed likely to have been selected, on the basis of the results, from multiple eligible analyses of the data?	N	N	PN	N	N	N	N	N
Comments			Results not reported in detail for all analyses					
Risk of bias in selection of the reported result	Low	Some concerns	Low	Some concerns	Some concerns	Some concerns	low	low
Overall risk of bias	High	High	High	High	High	high	high	high

First author (publication year)	Ebenfeld et al 2021	Zarski et al 2021	Stover et al 2022	Walter et al 2022	Wiemer et al 2022	Krämer et al 2022	Weise et al 2022	Zurowski et al 2023
DiGA	HB Panik	HB Vaginismus	Kalmeda	Kalmeda 2	Kranus Edera	Selfapy depression	vivira	invirto
Outcome (time of measurement) that is being assessed for risk of bias								
1.1 Was the allocation sequence random?	Y	Y	Y	NI	Y	Y	Y	NI
Comments								
1.2 Was the allocation sequence concealed until participants were enrolled and assigned to interventions?	NI	Y	NI	NI	NI	Y	NI	NI
Comments								
1.3 Did baseline differences between intervention groups suggest a problem with the randomization process?	NI	NI	N	N	NI	N	NI	N
Comments		No test for significance			No test for significance			
Risk of bias arising from the randomization process	Some concerns	low	Some concerns	Some concerns	Some concerns	Low	Some concerns	Some concerns
2.1. Were participants aware of their assigned intervention during the trial?	Y	Y	Y	PY	N	PY	Y	Y
Comments						Participants know about intervention they get.		

First author (publication year)	Ebenfeld et al 2021	Zarski et al 2021	Stover et al 2022	Walter et al 2022	Wiemer et al 2022	Krämer et al 2022	Weise et al 2022	Zurowski et al 2023
2.2. Were carers and people delivering the interventions aware of participants' assigned intervention during the trial?	N	Y	N	PY	N	Y	Y	Y
Comments								
2.3. If Y/PY/NI to 2.1 or 2.2: Were there deviations from the intended intervention that arose because of the trial context?	NI	NI	N	NI	NA	NI	NI	NI
Comments								
2.4 If Y/PY to 2.3: Were these deviations likely to have affected the outcome?	NA	NA	NA	NA	NA	NA	NA	NA
Comments								
2.5. If Y/PY/NI to 2.4: Were these deviations from intended intervention balanced between groups?	NA	NA	NA	NA	NA	NA	NA	NA
Comments								
2.6 Was an appropriate analysis used to estimate the effect of assignment to intervention?	Y	Y	N	PY	Y	Y	PY	Y
Comments								
2.7 If N/PN/NI to 2.6: Was there potential for a substantial impact (on the result) of the failure to analyse participants in the group to which they were randomized?	NA	NA	NI	NA	NA	NA	NA	NA
Comments								
Risk of bias due to deviations from the intended interventions	Some concerns	Some concerns	High	Some concerns	Low	Some concerns	Some concerns	Some concerns
3.1 Were data for this outcome available for all, or nearly all, participants randomized?	N	N	N	N	Y	N	N	N
Comments								
3.2 If N/PN/NI to 3.1: Is there evidence that the result was not biased by missing outcome data?	N	N	PN	PN	NA	PN	N	N

First author (publication year)	Ebenfeld et al 2021	Zarski et al 2021	Stover et al 2022	Walter et al 2022	Wiemer et al 2022	Krämer et al 2022	Weise et al 2022	Zurowski et al 2023
**Comments**								
**3.3 If N/PN to 3.2: Could missingness in the outcome depend on its true value?**	**Y**	**Y**	**Y**	**NI**	**NA**	**PY**	**PY**	**NI**
**Comments**								
**3.4 If Y/PY/NI to 3.3: Is it likely that missingness in the outcome depended on its true value?**	**NI**	**Y**	**NI**	**Y**	**NA**	**PY**	**NI**	**NI**
**Comments**								
**Risk of bias due to missing outcome data**	**high**	**high**	**high**	**high**	**low**	**high**	**high**	**high**
**4.1 Was the method of measuring the outcome inappropriate?**	**N**	**N**	**N**	**N**	**N**	**N**	**N**	**N**
**Comments**								
**4.2 Could measurement or ascertainment of the outcome have**	**N**	**N**	**PN**	**PN**	**N**	**N**	**N**	**N**

First author (publication year)	Ebenfeld et al 2021	Zarski et al 2021	Stover et al 2022	Walter et al 2022	Wiemer et al 2022	Krämer et al 2022	Weise et al 2022	Zurowski et al 2023
differed between intervention groups?								
Comments								
4.3 If N/PN/NI to 4.1 and 4.2: Were outcome assessors aware of the intervention received by study participants?	Y	Y	PY	Y	Y	PY	Y	Y
Comments								
4.4 If Y/PY/NI to 4.3: Could assessment of the outcome have been influenced by knowledge of intervention received?	Y	Y	PY	Y	Y	PY	Y	Y
Comments								
4.5 If Y/PY/NI to 4.4: Is it likely that assessment of the outcome was influenced by knowledge of intervention received?	NI	NI	NI	NI	NI	NI	NI	NI
Comments								
Risk of bias in measurement of the outcome	high	high	High	high	high	high	high	high
5.1 Were the data that produced this result analysed in accordance with a pre-specified analysis plan that was finalized before unblinded outcome data were available for analysis?	Y	Y	Y	NI	NI	Y	NI	NI
Comments				No study protocol	No study protocol		No study protocol	No study protocol
5.2 Is the numerical result being assessed likely to have been selected, on the basis of the results, from multiple eligible outcome measurements within the outcome domain?	N	N	N	N	N	N	N	N
Comments								
5.3 Is the numerical result being assessed likely to have been selected, on the basis of the results, from multiple eligible analyses of the data?	PN	N	N	PN	PN	PY	N	PN

First author (publication year)	Ebenfeld et al 2021	Zarski et al 2021	Stover et al 2022	Walter et al 2022	Wiemer et al 2022	Krämer et al 2022	Weise et al 2022	Zurowski et al 2023
Comments	Not all results of study completer analysis reported, yet nor relevant; missing analyses (PP, cost-effectiveness) compared to study protocol					Only results of pooling of imputation models reported. Not all results for all measurement points of analysis.		Results of ITT in text
Risk of bias in selection of the reported result	low	low	low	Some concerns	Some concerns	high	Some concerns	Some concerns
Overall risk of bias	high	high	high	high	high	high	high	high

First author (publication year)	Helbig Lang et al 2023	Baumeister & Moritz 2023	Gemesi et al 2023	Roth et al 2023	Rupp et al 2024	Rubel et al 2024
DiGA	Mindable	Novego Depression	Oviva	zanadio	NichtRaucherHeldenApp	Selfapy generalisierte Angststörung
Outcome (time of measurement) that is being assessed for risk of bias						
1.1 Was the allocation sequence random?	Y	Y	Y	NI	PY	Y
Comments						
1.2 Was the allocation sequence concealed until participants were enrolled and assigned to interventions?	Y	NI	Y	NI	PN	Y
Comments						
1.3 Did baseline differences between intervention groups suggest a problem with the randomization process?	PY	PN	N	NI	NI	PN
Comments						
Risk of bias arising from the randomization process	Some concerns	Some concerns	Low	Some concerns	high	low
2.1. Were participants aware of their assigned intervention during the trial?	Y	Y	Y	Y	Y	PN
Comments						
2.2. Were carers and people delivering the interventions aware of	N	PN	N	Y	N	N

First author (publication year)	Helbig Lang et al 2023	Baumeister & Moritz 2023	Gemesi et al 2023	Roth et al 2023	Rupp et al 2024	Rubel et al 2024
participants' assigned intervention during the trial?						
Comments						
2.3. If Y/PY/NI to 2.1 or 2.2: Were there deviations from the intended intervention that arose because of the trial context?	NI	NI	NI	NI	NI	NA
Comments						
2.4 If Y/PY to 2.3: Were these deviations likely to have affected the outcome?	NA	NA	NA	NA	NA	NA
Comments						
2.5. If Y/PY/NI to 2.4: Were these deviations from intended intervention balanced between groups?	NA	NA	NA	NA	NA	NA
Comments						
2.6 Was an appropriate analysis used to estimate the effect of assignment to intervention?	Y	PY	N	Y	PY	PY
Comments					ITT, yet only for primary outcome.	
2.7 If N/PN/NI to 2.6: Was there potential for a substantial impact (on the result) of the failure to analyse participants in the group to which they were randomized?	NA	NA	NI	NA	NA	NA
Comments						
Risk of bias due to deviations from the intended interventions	Some concerns	Some concerns	High	Some concerns	Some concerns	Low
3.1 Were data for this outcome available for all, or nearly all, participants randomized?	N	N	N	N	N	N
Comments						
3.2 If N/PN/NI to 3.1: Is there evidence that the result was not biased by missing outcome data?	PN	PY	N	Y	N	PN

First author (publication year)	Helbig Lang et al 2023	Baumeister & Moritz 2023	Gemesi et al 2023	Roth et al 2023	Rupp et al 2024	Rubel et al 2024
Comments						
3.3 If N/PN to 3.2: Could missingness in the outcome depend on its true value?	N	NA	NI	NA	NI	NI
Comments						No documentation of reasons for drop-outs
3.4 If Y/PY/NI to 3.3: Is it likely that missingness in the outcome depended on its true value?	NA	NA	NI	NA	NI	PY
Comments						High difference in drop-out rates, significant group differences
Risk of bias due to missing outcome data	Low	Low	High	Low	High	High
4.1 Was the method of measuring the outcome inappropriate?	N	N	N	N	N	N
Comments						

First author (publication year)	Helbig Lang et al 2023	Baumeister & Moritz 2023	Gemesi et al 2023	Roth et al 2023	Rupp et al 2024	Rubel et al 2024
4.2 Could measurement or ascertainment of the outcome have differed between intervention groups?	N	N	N	N	N	N
Comments						
4.3 If N/PN/NI to 4.1 and 4.2: Were outcome assessors aware of the intervention received by study participants?	Y	Y	NI	Y	Y	Y
Comments						
4.4 If Y/PY/NI to 4.3: Could assessment of the outcome have been influenced by knowledge of intervention received?	Y	Y	N	Y	Y	PY
Comments						
4.5 If Y/PY/NI to 4.4: Is it likely that assessment of the outcome was influenced by knowledge of intervention received?	NI	NI	NA	NI	NI	NI
Comments						
Risk of bias in measurement of the outcome	High	high	low	high	high	high
5.1 Were the data that produced this result analysed in accordance with a pre-specified analysis plan that was finalized before unblinded outcome data were available for analysis?	N	NI	NI	NI	NI	Y
Comments		No study protocol	No study protocol	No study protocol		
5.2 Is the numerical result being assessed likely to have been selected, on the basis of the results, from multiple eligible outcome measurements within the outcome domain?	N	Y	N	Y	Y	N
Comments						
5.3 Is the numerical result being assessed likely to have been	N	PN	Y	N	Y	N

First author (publication year)	Helbig Lang et al 2023	Baumeister & Moritz 2023	Gemesi et al 2023	Roth et al 2023	Rupp et al 2024	Rubel et al 2024
selected, on the basis of the results, from multiple eligible analyses of the data?						
Comments		Some results of analyses might be missing.				
Risk of bias in selection of the reported result	Some concerns	high	High	high	high	Low
Overall risk of bias	high	high	High	high	high	high

Part 2: RoB assessment of cluster RCT

First author (publication year)	Priebe et al 2022
DiGA	Kaia Rücken
Outcome (time of measurement) that is being assessed for risk of bias	
1a.1 Was the allocation sequence random?	Y
Comments	
1.a.2 Was the allocation sequence concealed until clusters were enrolled and assigned to interventions?	PY
Comments	
1.a.3 Did baseline differences between intervention groups suggest a problem with the randomization process?	NI
Comments	
Risk of bias arising from the randomization process	low
1b.1. Were all the individual participants identified and recruited (if appropriate) before randomization of clusters?	N
Comments	
1b.2 If N/PN/NI to 1b.1: Is it likely that selection of individual	PN

First author (publication year)	Priebe et al 2022
participants was affected by knowledge of the intervention assigned to the cluster?	
Comments	
1b.3 Were there baseline imbalances that suggest differential identification or recruitment of individual participants between intervention groups?	NI
Comments	
Risk of bias arising from the randomization process	low
2.1a Were participants aware that they were in a trial?	PN
Comments	
2.1b If Y/PY/NI to 2.1a: were participants aware of their assigned intervention during the trial?	NA
Comments	
2.2. Were carers and people delivering the interventions aware of participants' assigned intervention during the trial?	PY
Comments	
2.3. If Y/PY/NI to 2.1b or 2.2: Were there deviations from the intended intervention that arose because of the trial context?	PY
Comments	
2.4 If Y/PY to 2.3: Were these deviations likely to have affected the outcome?	PY
Comments	
2.5. If Y/PY/NI to 2.4: Were these deviations from intended intervention balanced between groups?	NI
Comments	
2.6 Was an appropriate analysis used to estimate the effect of assignment to intervention?	PN
Comments	

First author (publication year)	Priebe et al 2022
2.7 If N/PN/NI to 2.6: Was there potential for a substantial impact (on the result) of the failure to analyse participants in the group to which they were randomized?	NI
Comments	
Risk of bias due to deviations from the intended interventions	High
3.1a Were data for this outcome available for all clusters that recruited participants?	Y
Comments	
3.1b Were data for this outcome available for all, or nearly all, participants within clusters?	N
Comments	
3.2 If N/PN/NI to 3.1a or 3.1b: Is there evidence that the result was not biased by missing outcome data?	N
Comments	
3.3 If N/PN to 3.2: Could missingness in the outcome depend on its true value?	PY
Comments	
3.4 If Y/PY/NI to 3.3: Is it likely that missingness in the outcome depended on its true value?	PY

First author (publication year)	Priebe et al 2022
Comments	
Risk of bias due to missing outcome data	high
4.1 Was the method of measuring the outcome inappropriate?	N
Comments	
4.2 Could measurement or ascertainment of the outcome have differed between intervention groups?	N
Comments	
4.3a If N/PN/NI to 4.1 and 4.2: Were outcome assessors aware that a trial was taking place?	PN
Comments	outcomes were self-reported by patients, yet: patients had no information about other trial group
4.3b If Y/PY/NI to 4.3a: Were outcome assessors aware of the intervention received by study participants?	NA
Comments	
4.4 If Y/PY/NI to 4.3b: Could assessment of the outcome have been influenced by knowledge of intervention received?	NA

First author (publication year)	Priebe et al 2022
Comments	
4.5 If Y/PY/NI to 4.4: Is it likely that assessment of the outcome was influenced by knowledge of intervention received?	NA
Comments	
Risk of bias in measurement of the outcome	low
5.1 Were the data that produced this result analysed in accordance with a pre-specified analysis plan that was finalized before unblinded outcome data were available for analysis?	NI
Comments	
5.2 Is the numerical result being assessed likely to have been selected, on the basis of the results, from multiple eligible outcome measurements within the outcome domain?	PY
Comments	No raw data for measurement of outcomes with different scales at all measurement points; no data for measurement of single clusters provided
5.3 Is the numerical result being assessed likely to have been selected, on the basis of the results, from multiple eligible analyses of the data?	PY
Comments	
Risk of bias in selection of the reported result	High
Overall risk of bias	High

Supplementary Data 1: Data extraction sheet (Excel)

This supplementary data file provides detailed information extracted from all 23 DiGA approval studies included in the review. Extracted variables include study and population characteristics, primary and secondary outcomes, rationale for outcome selection, outcome domains, measurement instruments, self-report status, intervention duration, timing of post-intervention assessments, dropout rates, follow-up assessments, and reported effects including effect sizes.

Supplementary Data 2: Table 1 (Excel)

This table provides an overview of key characteristics of the 23 DiGA approval studies included in this review. The studies are ordered by DiGA category according to the BfArM DiGA directory, followed alphabetically by DiGA name.

Supplementary Data 3: Table 2 (Excel)

This table summarizes key characteristics and findings of the 23 DiGA approval studies included in this review. The studies are ordered by DiGA category according to the BfArM DiGA directory, followed alphabetically by DiGA name.
